# Noncoding and coding mechanisms of aging-related heart failure with preserved ejection fraction associated with thyroid dysfunction

**DOI:** 10.1242/dmm.052207

**Published:** 2025-11-25

**Authors:** Sankalpa Chakraborty, Olivia Sloan, Bryce Dickerson, Gourav Chakraborty, Shuang Li, Curren Bounds, Sophia Lemus, Caleb Hickman, J. Mauro Calabrese, Viswanathan Rajagopalan

**Affiliations:** ^1^ Department of Biological Sciences, Arkansas State University, Jonesboro, AR 72401, USA; ^2^ Arkansas Biosciences Institute at Arkansas State University, Jonesboro, AR 72401, USA; ^3^ Department of Pharmacology, RNA Discovery Center, Lineberger Comprehensive Cancer Center, University of North Carolina at Chapel Hill, NC 27599, USA; ^4^ Department of Biomedical & Anatomical Sciences, New York Institute of Technology College of Osteopathic Medicine at Arkansas State University, Jonesboro, AR 72401, USA

**Keywords:** Noncoding RNA, Heart failure, HFpEF, Thyroid hormones, Inflammation

## Abstract

Heart failure with preserved ejection fraction (HFpEF) is a lethal, heterogeneous, geriatric syndrome. Long noncoding RNAs (lncRNAs) constitute the majority of the functional mammalian transcriptome and are key regulators in complex pathophysiological processes. However, the roles of lncRNAs in aging HFpEF associated with thyroid hormone (TH) dysfunction are unclear. We used the well-established ZSF1 rat model to investigate early and severe age-related HFpEF in 5-, 13- or 20-months-old (mo) animals. Both serum THs significantly decreased in HFpEF in a temporal manner. Echocardiograms showed preserved cardiac function. Gravimetric and histologic analyses showed significant cardiac hypertrophy in HFpEF. Microarrays and RT-qPCR revealed that three lncRNAs were significantly increased predominantly in 13-mo HFpEF. Knockdown of lncRNA showed improvement in cell viability, which was further enhanced with T3 (active TH). Microarray analyses showed that two mRNAs were significantly altered in early HFpEF. We also identified previously unreported tissue and serum inflammatory cytokine markers in early and late HFpEF. Taken together, we have shown novel noncoding and coding markers in early- and/or late-aging-related hypothyroid HFpEF. Further studies may develop translatable diagnostic and therapeutic targets for HFpEF.

## Teaser

**Summary:** The authors have shown novel noncoding RNA markers and their mechanisms in aging-related early and/or late aging hypothyroid heart failure with preserved ejection fraction.

## INTRODUCTION

Heart failure (HF) is considered a global pandemic affecting ∼64 million people worldwide and ∼6.7 million people in the US (Savarese et al., 2023; Martin et al., 2024). By the year 2030, HF is estimated to cost ∼$69.8 billion (Savarese et al., 2023). The prevalence of HF is increasing, and this may be attributed to the increased aging (ageing) of the population, increasing prevalence of HF risk factors and comorbidities, improved treatment/survival following cardiovascular (CV) diseases, increased awareness and improvement in diagnostic precision, etc. (Teramoto et al., 2022; Savarese et al., 2023).

Heart failure with preserved ejection fraction (HFpEF) constitutes ∼50% of HF cases (Harper et al., 2018; Dunlay et al., 2017), with increasing prevalence over the past two decades (Gentile et al., 2022; Teramoto et al., 2022). HFpEF is characterized by HF with ≥50% left ventricular (LV) ejection fraction (EF). In many cases, it is also associated with abnormal LV diastolic function (Adamczak et al., 2020), and comorbidities/risk factors including hypertension, metabolic and thyroid dysfunction, advanced age, inflammation, obesity, diabetes mellitus and renal dysfunction (Messerli et al., 2017; Tsioufis et al., 2017; Davila et al., 2018; Schauer et al., 2020; Deichl et al., 2022). HFpEF remains a major public health concern with high morbidity and mortality rates in the USA and worldwide (Harper et al., 2018), and treatment options are limited. Compared to HF with reduced ejection fraction or HFrEF (symptomatic HF with LVEF ≤40%), older age is more strongly associated with HFpEF (its relative risk increased 90% per 10 years of age) (Ho et al., 2016).

Thyroid hormones (THs) are crucial CV regulators. Both T3 (triiodothyronine; active form) and T4 (thyroxine; prohormone) are secreted by the thyroid gland. Hypothyroidism is generally associated with low serum TH levels, decreased contractility and heart rate (HR), impaired diastolic relaxation, slowing of metabolic processes, dyslipidemia, inflammation, arrhythmia, etc. (Cappola et al., 2019; Rajagopalan and Gerdes, 2020, 2015; Gerdes and Ojamaa, 2016; Danzi et al., 2003). We and others have shown that THs may be decreased in serum and/or cardiac tissues in various forms of CV and associated disorders, and that optimal T3 dosing improved cardiac performance in hypothyroid cardiomyopathy, diabetic cardiomyopathy, hypertension, HF, myocardial infarction, ischemia-reperfusion injury, etc., without major adverse effects (Rajagopalan et al., 2020, 2023, 2017; Iervasi et al., 2003; Weltman et al., 2014, 2015; Pingitore et al., 2008).

With only ∼2% of the genome translating to proteins, the vast majority of the transcribable transcriptome comprises noncoding RNAs (ncRNAs). The majority (∼93%) of disease- and trait-associated variants emerging from genome-wide association studies were reported to lie within the noncoding regions (Maurano et al., 2012). Long noncoding RNAs (lncRNAs), which are defined as ncRNAs with more than 200 nucleotides, comprise the most functionally diverse class of ncRNAs (Kirk et al., 2018; Ma et al., 2013), and lncRNAs have been found to be critical for the development and progression of a variety of cardiovascular disorders. They have also been shown to be important in serving as biomarkers and therapeutic targets, and a few are in clinical trials (Das et al., 2020; Sallam et al., 2018; McKinsey et al., 2018). However, the role of aforesaid ncRNAs in the development of hypothyroidism (i.e. low TH levels), especially in the aging/aged HFpEF heart, is not clear.

This study explores the mechanistic landscape of HFpEF from multiple standpoints – aging, lncRNAs, mRNAs, thyroid dysfunction and inflammatory mediators. We used the hybrid ZSF1 [Zucker diabetic fatty (ZDF) spontaneously hypertensive heart failure (ZSF1)] obese rat model that presents with relevant features of clinical HFpEF including – but not limited to – diastolic dysfunction, HF, insulin resistance, hyperglycemia, hypertension, hyperlipidemia, exercise intolerance, etc. While the obese have been reported to have mutations in the leptin receptor [Lepr(fa), Lepr(cp)], the lean counterparts can be heterozygous and may serve as useful controls presenting with hypertension, but without HF or obesity (Schauer et al., 2020; Stolina et al., 2020). The findings presented in this study have not only shown transcripts associated with the early stage of HFpEF but also identified lncRNAs associated with aging and severity of the condition. In addition, this study also reports important pathways of coding RNAs along with multiple key markers of inflammation, which is a critical pathophysiological process in HFpEF.

## RESULTS

### ZSF1 obese HFpEF rats exhibit preserved contractile function

ZSF1 obese HFpEF rats at 5 months showed partially increased LV dimensions in systole and diastole compared to both WT and ZSF1 lean controls (Table 1). Nonetheless, both ejection fraction (in %) and fractional shortening (in %) were preserved in all the groups, especially the ZSF1 obese HFpEF rats, suggesting them to be a suitable model for HFpEF (in association with other HF signs discussed later). Our results also corroborate with previously reported findings, which showed a significant decrease in heart rate in 5-mo ZSF1 obese HFpEF males compared to ZSF1 lean counterparts (Nguyen et al., 2020; Tofovic et al., 2000). As characterized in other studies (Nguyen et al., 2020; Schauer et al., 2020), preliminary data showed that obese HFpEF rats also showed increased early mitral inflow velocity (E):early diastolic mitral annular velocity (E′) (E:E′ ratio) indicating diastolic dysfunction (Fig. S1).

**Table 1.** Preserved ejection fraction in hearts of male obese ZSF1 rats

	WT control (*n*=8)	Lean control (*n*=10)	Obese HFpEF (*n*=7)
Heart rate [bpm]	388.5±15.9	410.8±66.87	308±46.68
FS [%]	51.5±10.3	53.8±6.5	51±4.7
EF [%]	85.75±7	88.12±4	86.1±3
IVSd [cm]	0.15±0.02	0.18±0.02*	0.17±0.02
IVSs [cm]	0.3±0.05	0.34±0.03	0.33±0.04
LVIDd [cm]	0.69±0.03	0.78±0.09*	0.90±0.07****##
LVIDs [cm]	0.33±0.07	0.36±0.06	0.44±0.03**#
LVPWd [cm]	0.18±0.03	0.2±0.03	0.19±0.05
LVPWs [cm]	0.29±0.05	0.32±0.03	0.32±0.05

Comparison of heart function in wild-type (WT) control, ZSF1 lean control and ZSF1 obese HFpEF male rats.

Bpm, beats per minute; EF: ejection fraction; IVSd, interventricular septal dimension in diastole; IVSs, interventricular septal dimension in systole; FS: fractional shortening; HFpEF, heart failure with preserved ejection fraction; LVIDd, left ventricular internal diameter end diastole; LVIDs, left ventricular internal diameter end systole; LVPWd, left ventricular posterior wall diameter in diastole; LVPWs, left ventricular posterior wall diameter in systole. Statistical analyses were performed using one-way ANOVA with Tukey's multiple comparison test; means±standard deviation; *P<0.05; **P<0.01; ****P<0.0001 vs WT control; ^#^*P*<0.05; ^##^*P*<0.01 vs ZSF1 lean control.

### Serum TH levels are significantly decreased in both early and late HFpEF rats

**Fig. 1.**
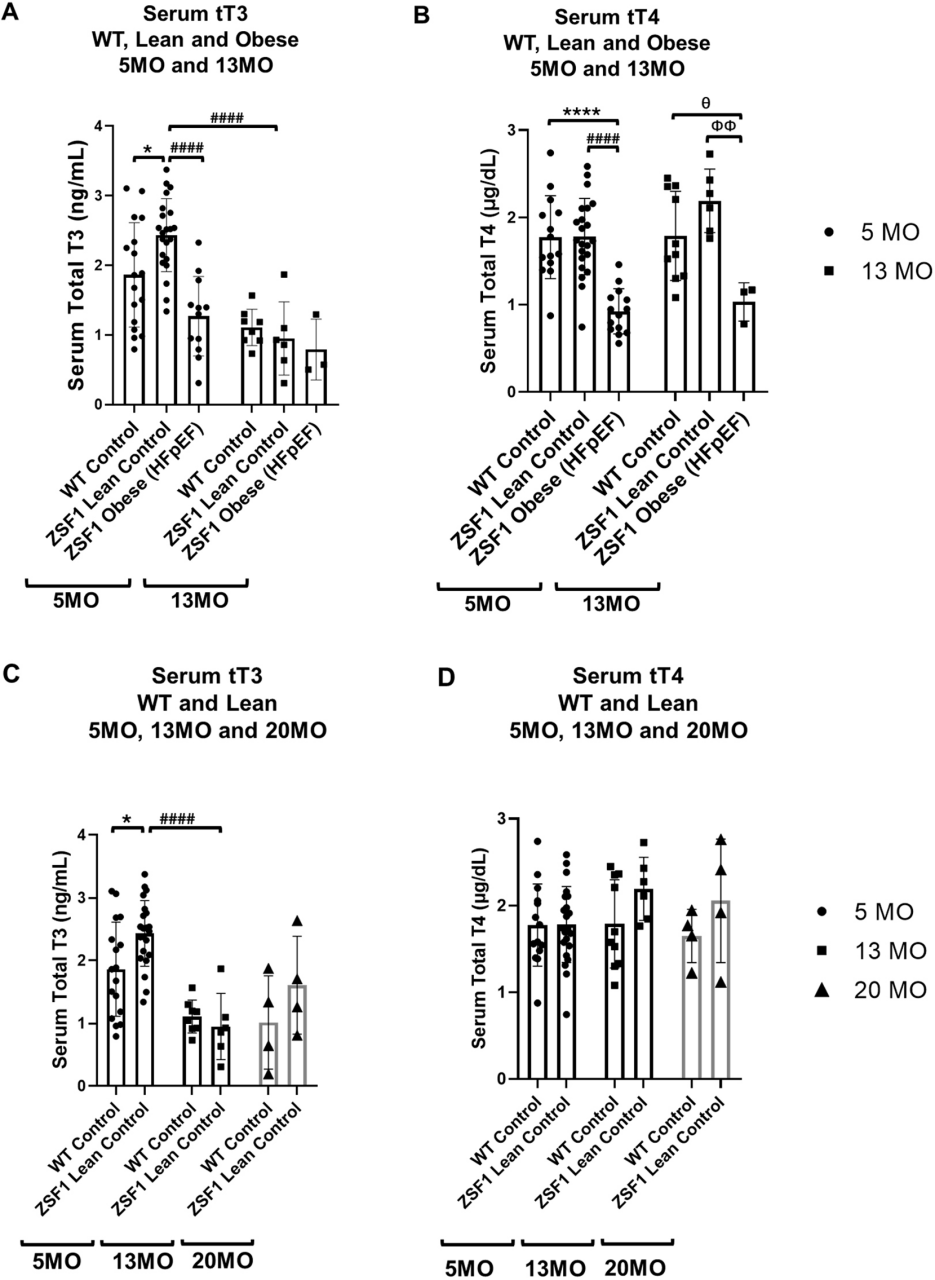
**Male rats with age-related HFpEF show persistent hypothyroidism.** (A,B) Serum total T3 (tT3; A) and total T4 (tT4; B) levels in 5-mo and 13-mo male WT control, ZSF1 lean control, and ZSF1 obese rats. (C,D) Serum tT3 (C) and tT4(D) in levels in 5-mo, 13-mo and 20-mo male WT control and ZSF1 lean control rats. *n*=19 (5 mo), *n*=13 (13 mo), *n*=8 (20 mo) in WT control, *n*=35 (5 mo), *n*=11 (13 mo), *n*=7 (20 mo) in Lean Control and *n*=12 (5 mo), *n*=3 (13 mo) in Obese HFpEF groups. All values were analyzed using two-way ANOVA (TWA) with Tukey's multiple comparison test and presented as means±standard deviation. WT, wild type; T4, thyroxine; T3, triiodothyronine. *P<0.05, ****P<0.0001 vs 5-mo WT control (TWA); ^####^*P*<0.0001 vs 5-mo ZSF1 lean control (TWA); ^ΦΦ^*P*<0.01 vs 13-mo ZSF1 lean control (TWA); ^θ^*P*<0.05 vs 13-mo obese HFpEF (*t*-test).

TH ELISA revealed that total serum T3 (Fig. 1A) and T4 (Fig. 1B) levels significantly decreased in 5-mo male ZSF1 obese HFpEF rats compared to male ZSF1 lean controls. In addition, serum T4 levels remained significantly lower in ZSF1 obese HFpEF rats compared to the ZSF1 lean controls even at the 13-mo timepoint (Fig. 1B). In females, total T3 (tT3) serum levels were significantly decreased in ZSF1 lean controls at both 13 months and 20 months compared to the 5 months timepoint (Fig. S2A). In males, tT3 serum levels were significantly lower in 13-mo vs 5-mo groups (Fig. 1C). In addition, based on two-way ANOVA, there was a significant (*P*<0.05) interaction effect between phenotype/genotype and age. Interestingly, total T4 (tT4) serum levels did not reveal any significant alterations among WT and ZSF1 lean controls over time in both males (Fig. 1D) and females (Fig. S2B). Taken together, the results indicate a significant and persistent hypothyroid phenotype associated with both early and late stages of the HFpEF pathology.

### ZSF1 obese HFpEF rats show hypertrophy of the heart and other systems

**Fig. 2.**
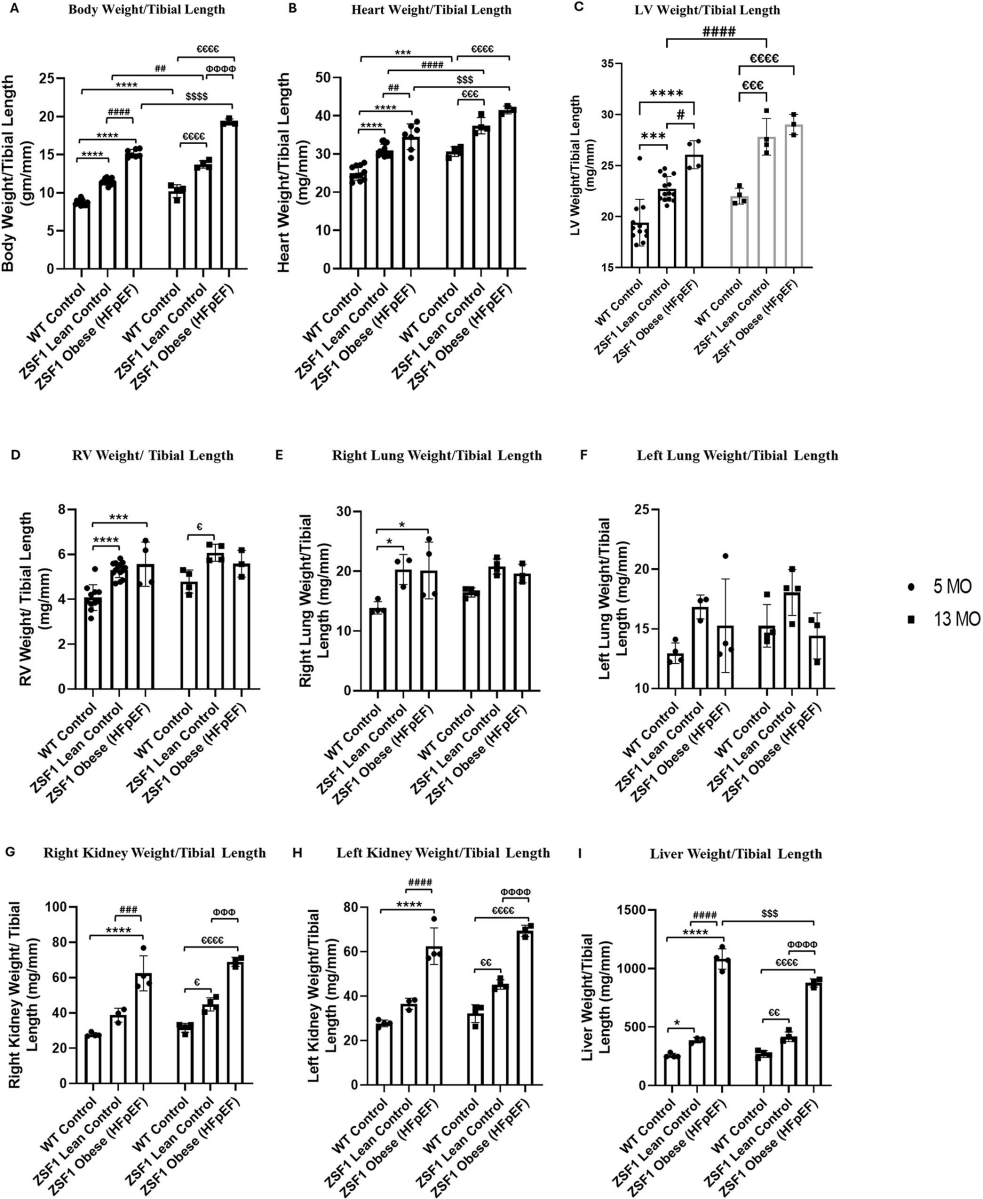
**Normalized gravimetrics in 5-mo and 13-mo male WT control, ZSF1 lean control and ZSF1 obese HFpEF rats.** (A) Body weight/tibial length, (B) heart weight/tibial length, (C) LV weight/tibial length, (D) RV weight/tibial length, (E) right lung weight/tibial length, (F) left lung weight/tibial length, (G) right kidney weight/tibial length, (H) left kidney weight/tibial length and (I) liver weight/tibial length. *n*=19 (5 mo), *n*=13 (13 mo), *n*=8 (20 mo) in WT control, *n*=35 (5 mo), *n*=11 (13 mo), *n*=7 (20 mo) in ZSF1 Lean Control and *n*=12 (5 mo), *n*=3 (13 mo) in ZSF1 Obese HFpEF groups. All values were analyzed using two-way ANOVA with Tukey's multiple comparison test and are presented as means±standard deviation. TH, thyroid hormone; WT, wild-type; LV, left ventricle; RV, right ventricle weight, *P<0.05, ***P<0.001, ****P<0.0001 vs 5-mo WT control; ^#^*P*<0.05, ^##^*P*<0.01, ^###^*P*<0.001, ^####^*P*<0.0001 vs 5-mo ZSF1 lean control; ^$$$^*P*<0.001, ^$$$$^*P*<0.0001 vs 5-mo ZSF1 obese (HFpEF); ^ε^*P*<0.05, ^εε^*P*<0.01, ^εεε^*P*<0.001, ^εεεε^*P*<0.0001 vs 13-mo WT control; ^ΦΦΦ^*P*<0.001, ^ΦΦΦΦ^*P*<0.0001 vs 13-mo ZSF1 lean control.

Normalized gravimetric analyses (Fig. 2A-I) showed significantly increased heart weights and LV weights, indicating cardiac hypertrophy in ZSF1 obese HFpEF rats (Fig. 2B,C). These findings were corroborated by the increase in the cardiomyocyte area of LVs in ZSF obese HFpEF, detected by histological staining (Fig. S5). Comparative analyses of 5-, 13- or 20-mo WT and ZSF1 lean controls (Fig. S3A-I) showed significant increases in LV weights in both males (Fig. S2C) and females (Fig. S4C), indicating induction of left ventricular hypertrophy in aged ZSF1 lean controls. This applies to relevant aged ZSF1 obese HFpEF groups as well (Fig. 2B,C). Since HFpEF is associated with comorbidities and other organs have been shown to be affected in early HFpEF, we studied them in our aged rats (Teramoto et al., 2022; Deichl et al., 2022; Hamdani et al., 2013). Comparison of male 5-, 13- and 20-mo WT vs ZSF1 lean controls showed significant increases in right lung weight (Fig. S3E), left kidney weights (Fig. S3H) and liver weight (Fig. S3I) in ZSF1 lean controls at all timepoints. For the heart, LV and left kidney, the normalized weights in ZSF1 lean controls were significantly increased at 13-mo compared to the 5-mo group (Fig. S3B,C,H). Furthermore, two-way ANOVA analyses of 5-mo and 13-mo WT control, ZSF1 lean control and obese HFpEF males showed a significant (*P*<0.001) interaction effect between phenotype/genotype and age, in both body weight and liver weight. Likewise, a comparison of 5-, 13- and 20-mo WT and ZSF1 lean controls also showed a significant (*P*<0.05) interaction effect between phenotype/genotype and age in body, heart and LV weights, in both females and males. Additionally, males also showed a significant (*P*<0.01) interaction effect in liver weight. Together, these findings have thrown new light on hypertrophy of the heart and other related organ systems in this ZSF1 model (Larson et al., 2022).

### Significant differentially expressed lncRNAs in HFpEF hearts

**Fig. 3.**
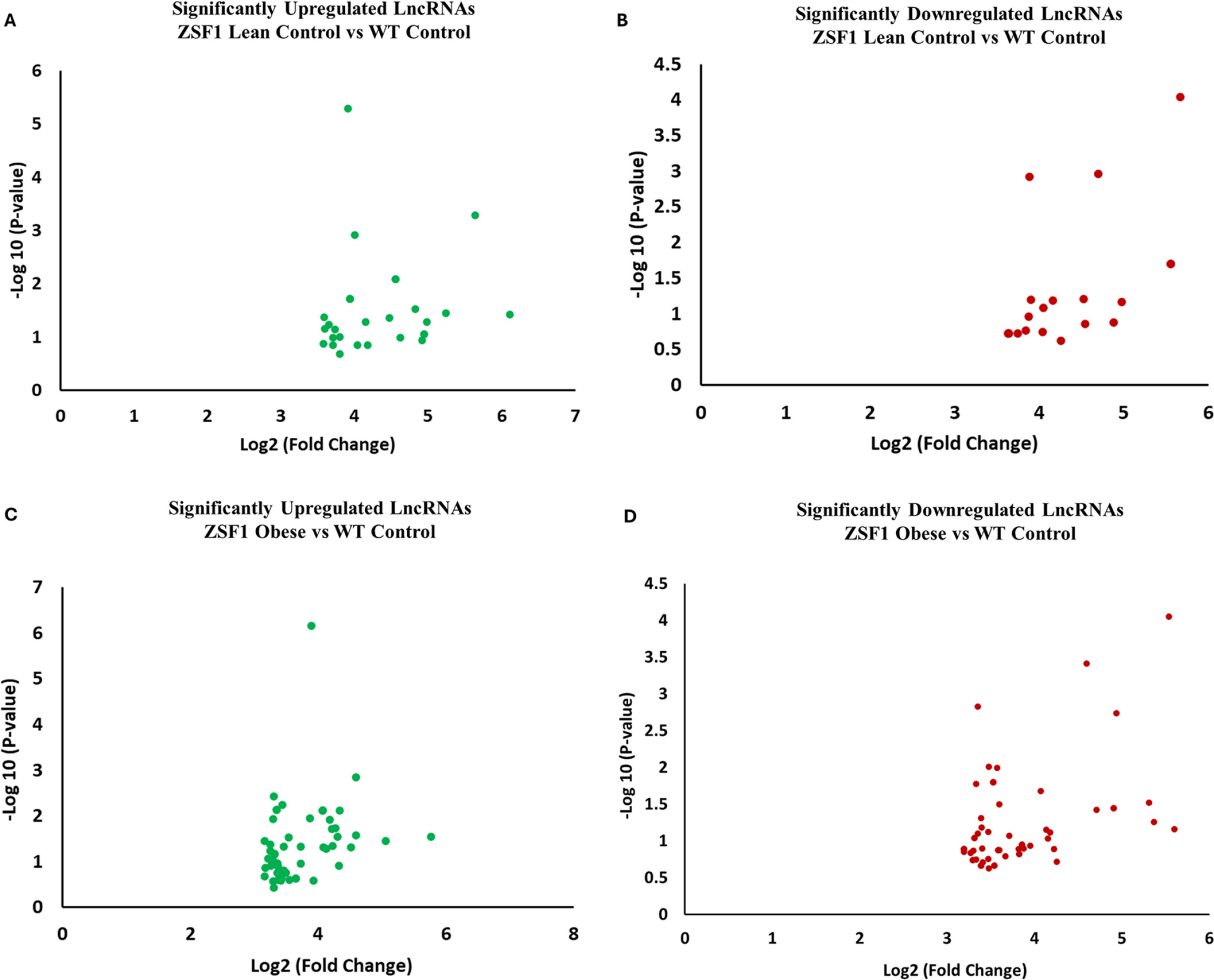
**Differentially expressed LV lncRNAs in HFpEF rats.** Microarray analyses of lncRNA (*P*<0.05) and further refinement (fold change of >1.5 and FDR<0.05) of differentially expressed lncRNAs in 5-mo WT control, ZSF1 lean control and ZSF1 obese rats (*n*=4/group). (A) Upregulated lncRNAs in ZSF1 lean vs WT controls. (B) Downregulated lncRNAs in ZSF1 lean vs WT controls. (C) Upregulated lncRNAs in ZSF1 obese (HFpEF) vs WT controls. (D) Downregulated lncRNAs in ZSF1 obese (HFpEF) vs WT controls. Detailed statistical analyses are elaborated in the Materials and Methods section.

The analyses showed that several unique lncRNAs were significantly altered [*P*<0.05; >1.5-fold change with false discovery rates (FDR)<0.05] in WT control LVs compared to the ZSF1 lean control group or compared to the ZSF1 obese HFpEF group. We found 29 upregulated (Fig. 3A) and 20 downregulated (Fig. 3B) lncRNAs in the ZSF1 lean control group compared to the WT controls. Furthermore, we found 57 upregulated (Fig. 3C) and 61 downregulated (Fig. 3D) lncRNAs in ZSF1 obese HFpEF vs WT control comparison. In addition, 12 upregulated and 12 downregulated unique lncRNAs were also found to be common between 5-mo ZSF1 lean vs WT control groups and obese HFpEF vs WT control groups (see Table S1, marked in red). Table S1 also lists additional details, such as sequence identifier (seqname), gene symbol, RNA length, chromosome number, strand, transcription start site (txStart), transcription end site (txEnd), EntrezID, noncoding RNA characteristic, information about any associated genes, etc.

### Left ventricular mRNA microarray analyses

#### Significant differentially expressed mRNAs in HFpEF hearts

**Fig. 4.**
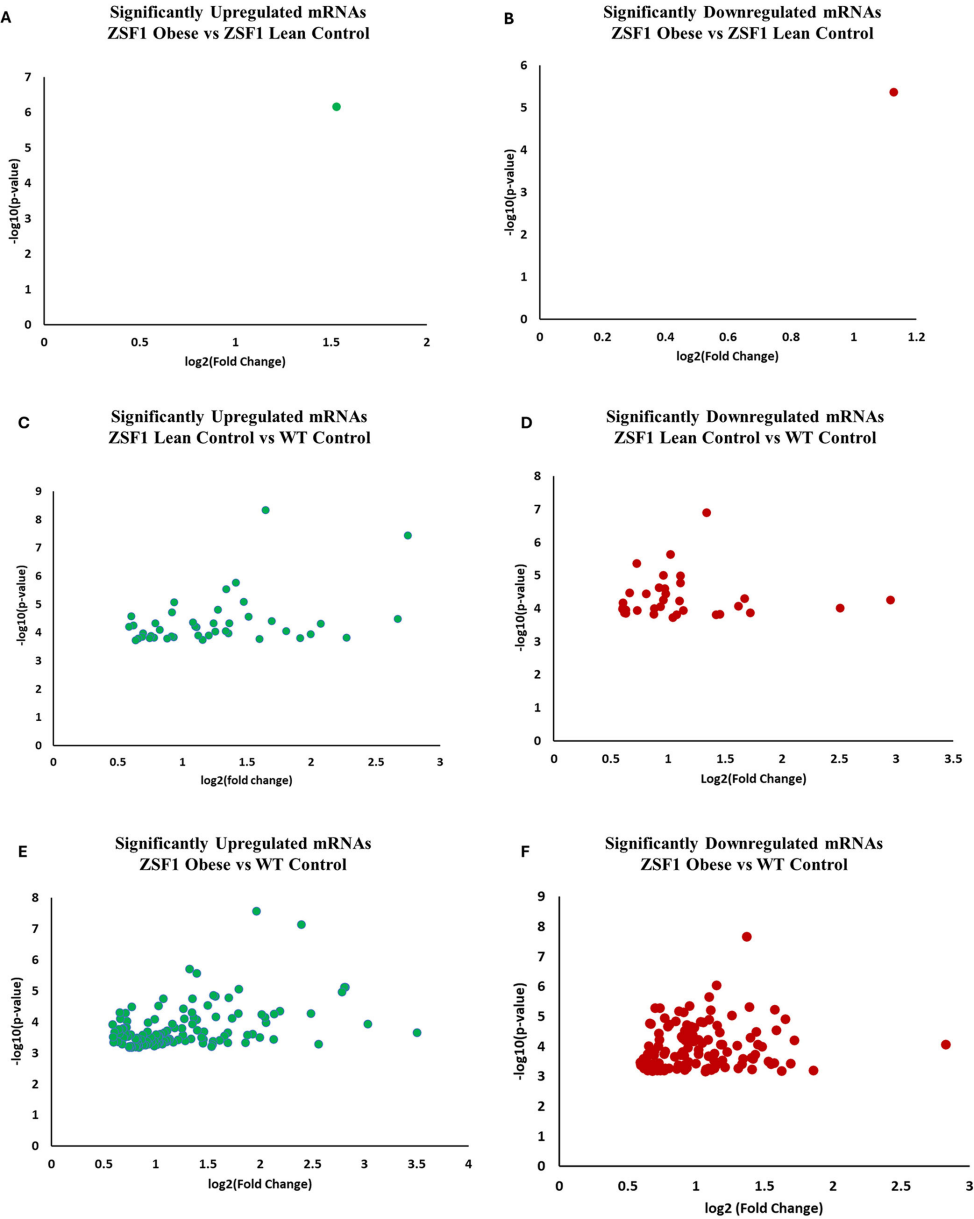
**Differentially expressed LV mRNAs in HFpEF rats.** mRNA microarray analyses (*P*<0.05) and further refinement (fold change >1.5 and FDR<0.05) identified differentially expressed mRNAs in 5-mo WT control, ZSF1 lean control and ZSF1 obese rats (*n*=4/group). (A) Upregulated mRNAs in ZSF1 obese (HFpEF) vs lean controls. (B) Downregulated mRNAs in ZSF1 obese (HFpEF) vs lean controls. (C) Upregulated mRNAs in ZSF1 lean vs WT controls. (D) Downregulated mRNAs in ZSF1 lean vs WT controls. (E) Upregulated mRNAs in ZSF1 obese (HFpEF) vs WT controls. (F) Downregulated mRNAs in ZSF1 obese (HFpEF) vs WT control. Detailed statistical analyses are elaborated in the Materials and Methods section.

mRNA microarray analysis showed numerous mRNAs that were differentially regulated (at least 1.5-fold with *P*-value and FDR<0.05) (Table S2) in ZSF1 groups in comparison with WT controls. The ZSF1 lean vs WT control comparison identified 43 upregulated (Fig. 4C) and 33 downregulated (Fig. 4D) mRNA transcripts. On the other hand, ZSF1 obese HFpEF and WT control showed 119 upregulated (Fig. 4E) and 122 downregulated (Fig. 4F) mRNAs. Differential expression analysis in ZSF1 lean and ZSF1 obese HFpEF comparison showed only one upregulated (*Sik1*) (Fig. 4A) and one downregulated (*Anxa13*) (Fig. 4B) mRNA transcript.

In addition, among the up- and down-regulated mRNAs in ZSF1 lean vs WT control comparisons, 26 upregulated and 21 downregulated unique mRNA transcripts were also up- or down-regulated in ZSF1 obese HFpEF vs WT control comparisons (*P*<0.05; FDR<0.05) (Table S2). This indicates that several genes from the non-HFpEF (lean) phenotype continue to be involved in the HFpEF (obese) phenotype.

#### mRNA enrichment analyses

##### Gene Ontology enrichment analyses

In Gene Ontology (GO) enrichment analyses (Table S3), ZSF1 lean control vs WT control showed four Biological Process (BP) pathway enrichments and three Cellular Component (CC) enrichments among the significantly upregulated genes (*P*<0.05) (Fig. S6B). Within the downregulated genes in ZSF1 lean control vs WT control, we found eight BP, one CC and one Molecular Function (MP) enrichments (*P*<0.05) (Fig. S6C). Groupwise comparison of ZSF1 obese HFpEF and WT control showed 21 GO-enriched BPs in significantly upregulated coding genes (Fig. S6A). Interestingly, a closer look of the BP enrichments showed distinct pathway clusters among the two aforesaid comparisons. BP enrichment of significantly upregulated mRNAs in obese HFpEF vs WT control comparison is predominantly a cluster of metabolic pathway genes, whereas BP enrichment of upregulated mRNAs in ZSF1 lean control vs WT control is a cluster of only immune response pathways. Furthermore, BP enrichment analyses of significantly downregulated mRNAs in ZSF1 lean control vs WT control showed a combination of oxygen/gas transport, metabolic pathways, immune response pathways and others.

##### KEGG pathway enrichment analyses

As listed in Table S4, the lean vs WT control comparison yielded two vector-borne disease pathways – malaria and African trypanosomiasis (Fig. S7C; *P*<0.05). The obese HFpEF showed upregulation of three different fatty acid pathways – fatty acid elongation, fatty acid metabolism and biosynthesis of unsaturated fatty acids compared to WT controls (Fig. S7D; *P*<0.05). On the other hand, the estrogen receptor pathway and circadian entrainment pathway were significantly downregulated in the ZSF1 obese HFpEF vs ZSF1 lean control comparison (Fig. S7B; *P*<0.05). This obese HFpEF vs lean control comparison also showed upregulation of five pathways including fatty acid elongation, biosynthesis of unsaturated fatty acids, fatty acid degradation, fatty acid metabolism and lysine degradation (Fig. S7A; *P*<0.05).

### HFpEF hearts show lncRNA−mRNA association

Similar to results from the above differentially expressed lncRNAs, further analyses showed that several lncRNAs had associated mRNAs (coding gene pairs) with a *P*<0.05. However, further sorting with an FDR<0.05 (Table S5), showed two potential lncRNA−mRNA associations in the ZSF1 obese HFpEF vs WT control group comparison. None of the other groups showed any significant lncRNA−mRNA associations. *LOC102548445* and *LOC102556567*, two significantly upregulated long intergenic noncoding RNAs (lincRNAs), were found to be closely associated with two mRNAs, respectively – *Ifi44l* and *AABR06107100.1*. While *Ifi44l* was downregulated, the X-chromosomal gene *AABR06107100.1* (likely associated with *Nxf5*) was upregulated in our mRNA differential expression analyses of obese HFpEF vs WT control (Table S5). We have also investigated the antisense lncRNAs and associated coding gene pairs but no groupwise comparisons showed any significantly altered antisense lncRNA−mRNA pairs after sorting (*P*<0.05, FDR<0.05).

### Real-time quantitative PCR identifies unique lncRNAs in older-age phenotypes

**Fig. 5.**
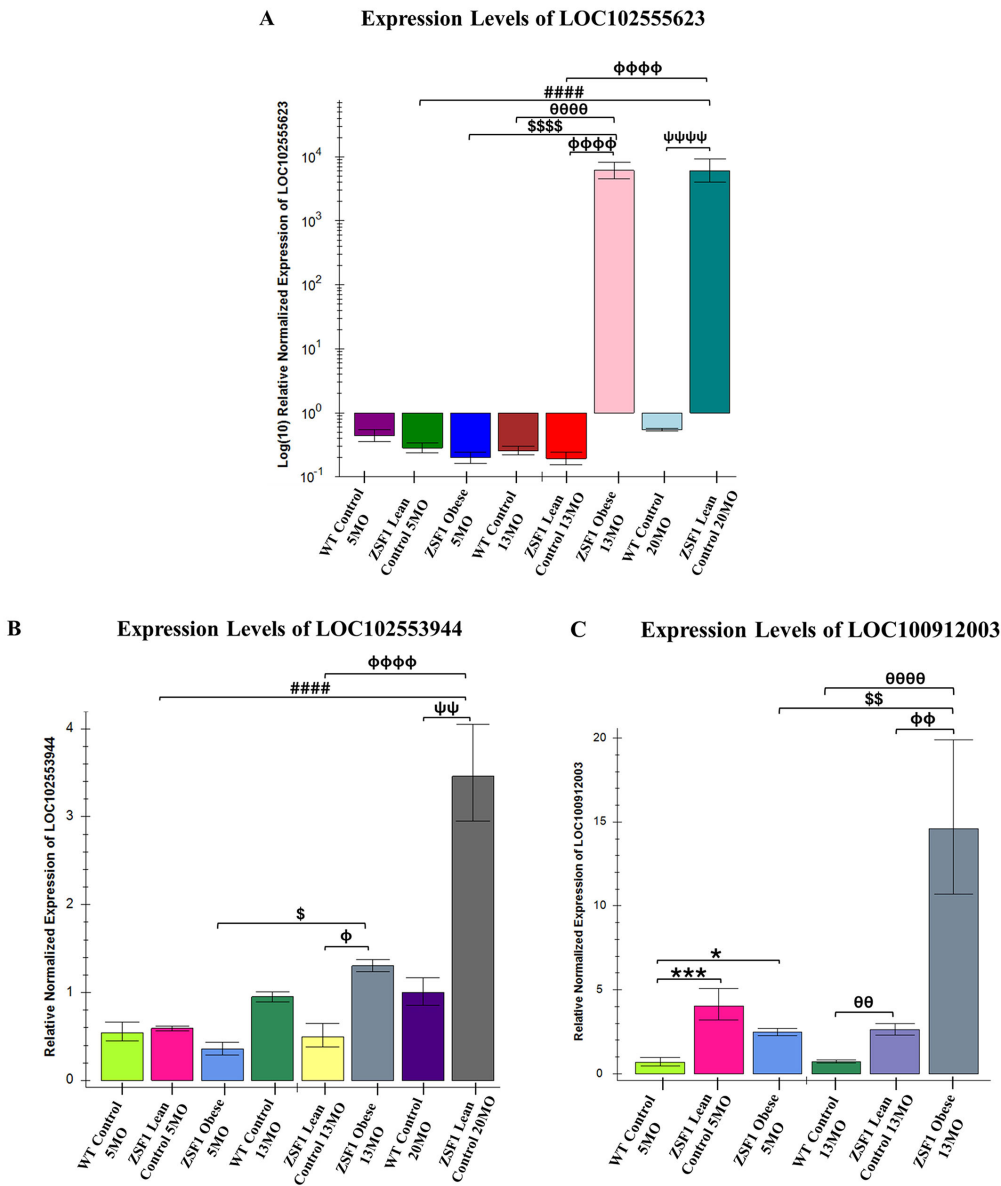
**RT-qPCR analyses identified novel lncRNAs associated with aging aging-related HFpEF and hypothyroidism in obese ZSF1 rats.** (A-C) RT-qPCR of 5-mo, 13-mo±20-mo WT control, ZSF1 lean control and ZSF1 obese HFpEF left ventricular (LV) samples showed significantly altered levels of *LOC102555623* (A), (B) *LOC102553944* (B) and (C) *LOC100912003* (C). RT-qPCR experiments were done in *n*=3-4 per group (biological replicates) and *n*=2 (technical replicates). Statistical analyses were performed using one-way ANOVA with Tukey's multiple comparison test. Results are presented as means±standard error. *P<0.05, ***P<0.001 vs 5-mo WT control; ^####^*P*<0.0001 vs 5-mo ZSF1 lean control; ^$^*P*<0.05, ^$$^*P*<0.01, ^$$$$^*P*<0.0001 vs 5-mo ZSF1 obese HFpEF; ^θθ^*P*<0.01, ^θθθθ^*P*<0.0001 vs 13-mo WT control; ^Φ^*P*<0.05, ^ΦΦ^*P*<0.01, ^ΦΦΦΦ^*P*<0.0001 vs 13-mo ZSF1 lean control; ^ΨΨ^*P*<0.01, ^ΨΨΨΨ^*P*<0.0001 vs 20-mo WT control.

As discussed earlier, preclinical HFpEF studies commonly focus on the early HFpEF timepoint (∼5 mo). As aging is strongly correlated with the development of HFpEF (Larson et al., 2022), we aimed to detect whether the lncRNA expression patterns change with age/severity of HFpEF pathologies. In order to identify aging-related HFpEF-associated lncRNAs, we performed RT-qPCR with select lncRNA targets from the microarray results on samples from all possible and available time points. To ensure high rigor in lncRNA microarray results, we did not include those targets that showed FDR>0.05 (even though *P*<0.05). We chose the top three to four targets from microarray analyses based on the highest fold change (*P*<0.05 and FDR<0.05) and excluded any genes suspected to be protein-coding or pseudogene. The analyses showed that 13-mo ZSF1 obese HFpEF rats had significantly higher expression levels of cardiac *LOC102555623* (Fig. 5A), *LOC102553944* (Fig. 5B) and *LOC100912003* (Fig. 5C) lncRNAs compared to the ZSF1 lean 13-mo group. The expression levels of both *LOC102555623* and *LOC100912003* were also found to be significantly increased in the 13-mo ZSF1 obese group compared to both the ZSF1 obese 5-mo and WT 13-mo groups. Interestingly, the 20-mo ZSF1 lean LVs had a similar level of *LOC102555623* expression compared to the 13-mo ZSF1 obese group. Furthermore, both *LOC102555623* and *LOC102553944* lncRNA expression levels were significantly increased in the ZSF1 lean 20-mo group compared to WT 20-mo and ZSF1 lean 13 mo mice but not when compared to obese HFpEF 13-mo mice. Additionally, the expression levels of these three lncRNAs showed similar trends in microarray and RT-qPCR among groups at 5 months. These indicate that the late-stage aged ZSF1 lean 20-mo rats develop molecular changes similar to the HFpEF group.

We identified one significantly upregulated (*Sik1*) and one downregulated mRNA (*Anxa13*) among the 5-mo ZSF1 lean control vs obese comparisons in the microarray analyses, RT-qPCR showed no significant changes during the late stage of the disease. This indicates the possibility that these genes may be critical during the onset of HFpEF rather than the progression or severity of the disease.

### Human lncRNAs exist in regions syntenic to several of the differentially expressed rat lncRNAs

Our analyses showed that 55 of the differentially expressed lncRNAs from our microarray data harbored at least one human GENCODE (https://www.gencodegenes.org/) lncRNA in their corresponding syntenic regions of hg38 (Table S6). Of these 55 lncRNAs, 14 harbored significant levels of non-linear similarity to at least one hg38 lncRNA in their corresponding syntenic regions, as assessed by the *k-mer*-based SEEKR algorithm (https://github.com/CalabreseLab/seekr). Between the obese HFpEF vs WT control comparison, we identified one upregulated (XR_343833.2) and eight downregulated rat lncRNAs (*uc.398+*, *ENSRNOT00000071067*, *XR_146205.6*, *XR_339540.1*, *XR_347235.3*, *XR_590684.2*, *XR_591291.2* and *XR_592571.4*). Their corresponding human counterparts are *ENSG00000307586*, *ENSG00000300045*, *ENSG00000274080*, *SNHG20*, *ENSG00000300534*, *ENSG00000259135*, *LINC01089*, *ENSG00000301039* and *ENSG00000309593*, respectively. Between the ZSF1 lean control vs WT control comparison, we identified one upregulated (*XR_592813.2*) and four downregulated rat lncRNAs (*XR_592571.4*, *XR_146205.6*, *XR_338611.3* and *XR_591291.2*). Their corresponding human counterparts are *GFOD3P*, *ENSG00000309593*, *SNHG20*, *CARINH* and *ENSG00000301039*, respectively. Three of the rat lncRNAs that were downregulated in both ZSF1 obese HFpEF and lean controls compared to the WT controls had human counterparts, namely *ENSG00000309593*, *SNHG20* and *ENSG00000301039*.

### Improved cell viability following *in vitro* inhibition of aging-related HFpEF-associated lncRNAs

**Fig. 6.**
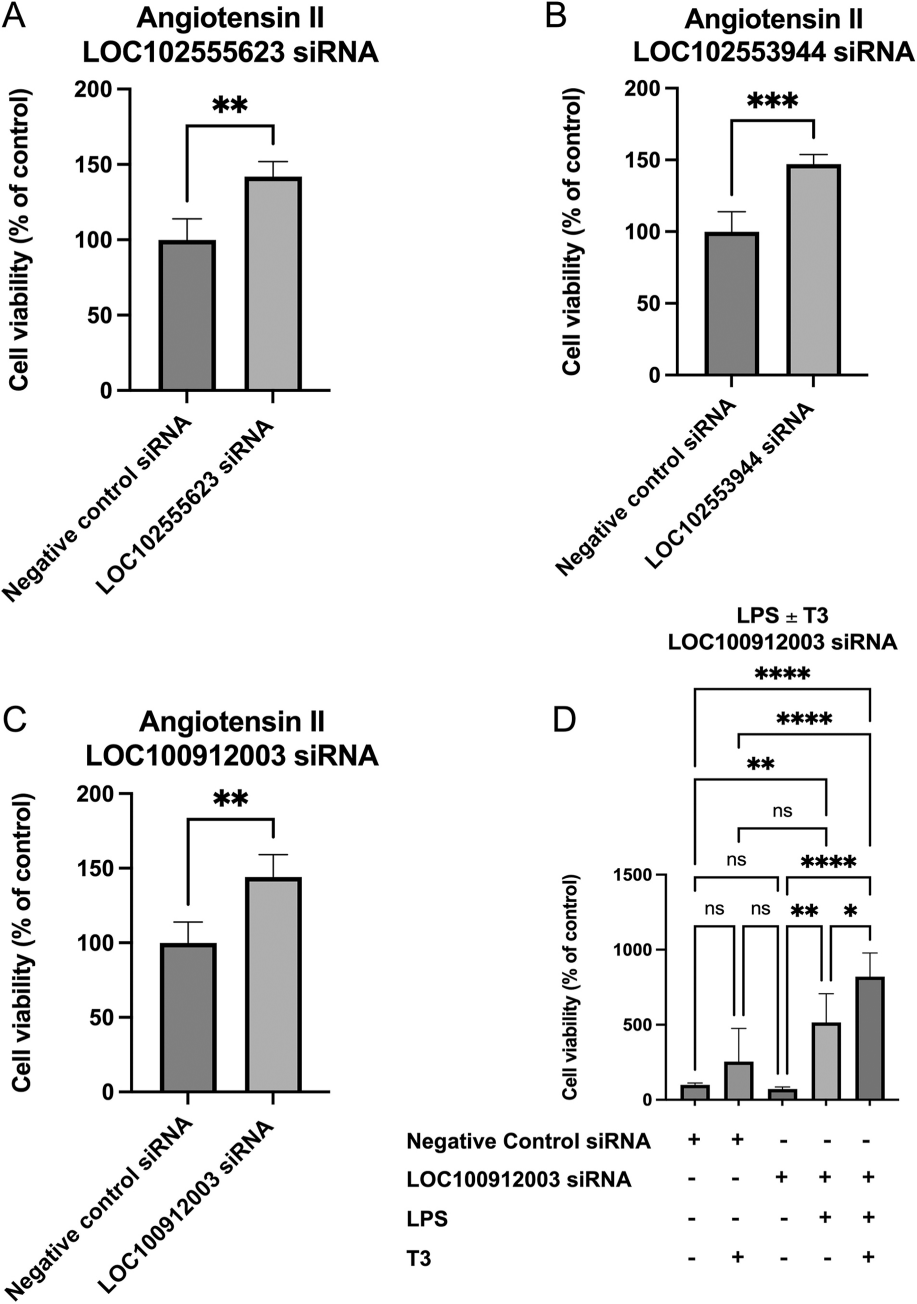
**Improvement in cell viability following siRNA knockdown of aging-related hypothyroid HFpEF-associated lncRNAs.** Cell viability assay showed a significant (*P*<0.05) increase in cell survival following knockdown of (A) *LOC102555623*, (B) *LOC102553944* or (C) *LOC100912003* in H9c2 rat cardiac myoblasts treated with 100 nM angiotensin II. H9c2 cells under lipopolysaccharide (LPS) stress (5 µg/ml), in the presence or absence of T3 (10 µM) also showed significant (*P*<0.05) improvement in cell survival following knockdown of (D) *LOC100912003*. Statistical analyses were performed using one-way ANOVA with Tukey's multiple comparison test. Results are presented as means±standard error (of quadruplicates). *P<0.05, **P<0.01, ***P<0.001, ****P<0.0001.

In angiotensin II (Ang II)-treated rat myoblast H9c2 cells, we found significantly (*P*<0.05) improved cell viability following siRNA-mediated downregulation of all the three aging-related HFpEF-associated lncRNAs identified via microarray and qPCR (Fig. 6A-C). Furthermore, in lipopolysaccharide (LPS)-treated cells, we found significant (*P*<0.05) improvements in cell viability when *LOC100912003* was inhibited (Fig. 6D). Importantly, we found that additional T3 (Sacripanti et al., 2018) treatment along with siRNA knockdown of *LOC100912003* under LPS stress further significantly improved the cell viability (Fig. 6D). These results indicate that aging-related HFpEF-associated lncRNAs are functionally important in cardiac cells. We also have uncovered a previously unexplored TH-lncRNA regulatory axis (Fig. 6D).

### Aging-related HFpEF shows altered serum and cardiac inflammatory markers

**Fig. 7.**
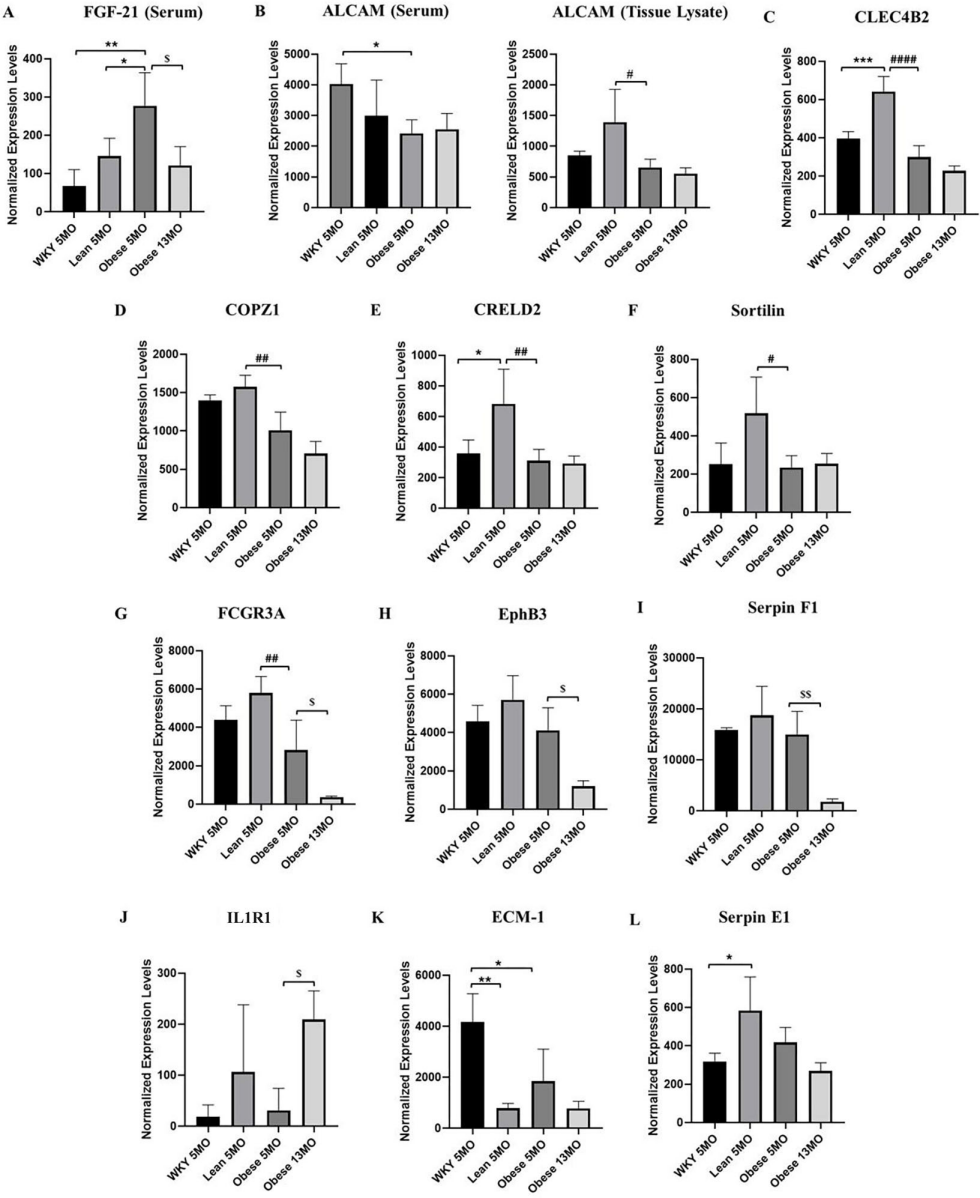
**Rats with aging-related HFpEF show altered serum and cardiac tissue inflammatory profiles.** Serum and cardiac tissue inflammatory factor profiles in 5-mo WT control, 5-mo ZSF1 lean control, 5-mo ZSF1 obese HFpEF and 13-mo ZSF1 obese HFpEF: (A) FGF21 (Serum); (B) ALCAM (Left subset: Serum; Right subset: Tissue Lysate); (C) CLEC4B2 (Tissue); (D) COPZ1 (Tissue); (E) CRELD2 (Tissue); (F) Sortilin (Tissue); (G) FCGR3A (Tissue); (H) EphB3 (Tissue) (I) Serpin F1 (Tissue); (J) IL1R1 (Tissue); (K) ECM-1 (Tissue); (L) Serpin E1 (Tissue). *n*=3 in WT (5 mo), *n*=4 in Lean Control (5 mo) and *n*=5 (5 mo), and *n*=3 (13 mo) in Obese HFpEF groups. The extracted raw data from quadruplicated assays were grouped and statistically analyzed using one-way ANOVA with Tukey's multiple comparison test. All values are presented as means±standard deviation. *P<0.05, **P<0.01, ***P<0.001 vs 5-mo WT control; ^#^*P*<0.05, ^##^*P*<0.01, ^####^*P*<0.0001 vs 5-mo ZSF1 lean control; ^$^*P*<0.05, ^$$^*P*<0.01 vs 5-mo ZSF1 obese HFpEF.

Chronic low–grade inflammation has been previously reported to be associated with HFpEF (Mesquita et al., 2021). Previous work from our lab (Rajagopalan et al., 2023) showed dysregulation of inflammatory pathway genes under altered TH levels. As hypothyroidism is a characteristic feature of HFpEF (Neves et al., 2020), we tried to identify clinically relevant inflammatory cytokine markers associated with the early and late stages of HFpEF. Moreover, significant alterations in inflammatory pathway genes in our GO analyses primed our interest to further investigate their protein profiles. Inflammation arrays in 5-moWT control, ZSF1 lean control and ZSF1 obese HFpEF, and 13-mo ZSF1 obese HFpEF rats showed markedly different trends in inflammatory marker levels in both LV tissue lysates and in serum (Fig. 7A-L; *P*<0.05). A majority of the alterations in inflammatory markers was observed in the LV tissue lysates.

Compared to lean rats, 5-mo (early) HFpEF showed a significant increase in serum FGF21 levels. This indicates that serum FGF21 may be used as a potential biomarker to detect early onset of HFpEF (discussed later). LV tissue levels of activated leukocyte cell adhesion molecule (ALCAM), C-type lectin domain family 4 member B2 (CLEC4B2), COPI coat complex subunit zeta 1 (COPZ1), CRELD disulfide isomerase 2 (CRELD2, also known as cysteine-rich with EGF-like domains 2) and Sortilin were significantly downregulated in 5-mo ZSF1 obese hearts compared to the ZSF1 lean controls and remained low in the 13-mo obese rats. Serum ALCAM levels showed a significant decrease in the 5-mo obese rats compared to age-matched WT controls. The Fc gamma receptor IIIa (FCGR3A; also known as CD16), not only showed a significant reduction in 5-mo obese LVs compared to the 5-mo lean counterparts (similar to the tissue FGF21) but was also further significantly reduced in the 13-mo obese HFpEF group. Interestingly, the tissue levels of EPH receptor B3 (EphB3) and serpin family F member 1 (SERPINF1, hereafter referred to as Serpin F1) were not significantly decreased in obese rats at the 5-mo time point compared to the controls but reduced significantly in late (13 mo) obese hearts compared to the 5-mo obese group. This indicates that EphB3 and Serpin F1 have the potential to serve as inflammatory markers to identify the severity of HFpEF. However, while tissue levels of IL1R1 did not show any significant difference between 5-mo lean vs 5-mo obese comparisons, it was significantly increased in 13-mo obese rats compared to the 5-mo obese rats. This indicates that, similar to EphB3 and Serpin F1, IL1R1 may also serve as a tissue biomarker to detect the severity of HFpEF. We found extracellular matrix protein 1 (ECM-1) levels were significantly reduced in both 5-mo ZSF1 lean controls and obese rats, indicating that it may serve as a biomarker for hypertension. Furthermore, similar to CLEC4B2 and CRELD2, levels of serpin family E member 1 (SERPINE1, hereafter referred to as Serpin E1) were significantly increased in 5-mo ZSF1 lean controls compared to 5-mo WT controls. These findings indicate the potential for the development of multiple biomarkers for HFpEF and/or hypertension.

## DISCUSSION

To our knowledge, this is the first preclinical study investigating the later stages of HFpEF. Compared to the ZSF1 lean controls, we uncovered that serum T4 levels were not only significantly decreased in the 5-mo obese HFpEF rats but also remained low in the 13-mo late HFpEF stage. This model provides a unique opportunity to shed light on possible mechanisms seen in human HFpEF with other comorbidities (Neves et al., 2020; Nguyen et al., 2020; Abdellatif et al., 2021; Adams et al., 2022; Silva et al., 2021; Moreira-Costa et al., 2024; Hamdani et al., 2013). This is also the first known report studying the effects of the ZSF1 phenotype for the longest possible duration (20 months). The role of lncRNAs in HFpEF as a geriatric syndrome with thyroid dysfunction has been unclear (Upadhya et al., 2017). Five months is the earliest timepoint where HFpEF is commonly studied (Hamdani et al., 2013). In support of detecting a broad array of targets that may help with preventive screening (in future studies) of potential biomarkers associated with aged hypothyroid HFpEF, we conducted the microarray at the 5-mo timepoint and compared the result with select top hit targets at the 13-mo timepoint using RT-qPCR. Accordingly, using lncRNA-specific microarray and subsequent RT-qPCR analyses, we discovered three novel lncRNAs. Levels of *LOC102555623*, *LOC102553944* and *LOC100912003* sequences were significantly elevated in the 13-mo obese HFpEF LVs compared to their 5-mo obese counterparts. In addition, although all three lncRNAs were not significantly altered in the 5-mo obese groups compared to the 5-mo lean groups, levels of all three were significantly higher in 13-mo obese groups compared to 13-mo lean groups. Furthermore, our findings that the levels of these lncRNAs in the 20-mo ZSF1 lean rats are either equally high or much higher than the 13-mo ZSF1 obese rats, suggest that the 20-mo ZSF1 lean rats are approaching a HF-like phenotype at this timepoint.

Search of quantitative trait loci (Vedi et al., 2023) in the Rat Genome Database showed that *LOC102553944* is significantly associated with multiple relevant traits, including body weight gain, feed conversion ratio, plasma corticosterone level, plasma insulin level, pancreas wet weight and plasma free fatty acids levels. In addition, *LOC102555623* was associated with systolic blood pressure, retroperitoneal fat pad weight to body weight ratio abdominal subcutaneous fat pad weight, lean tissue morphological measurement, abdominal fat pad weight to body weight ratio and both kidneys wet weight to body weight ratio. Further investigation can be helpful in understanding the pathophysiological roles, diagnostic potential and druggability of these three lncRNAs for aging-related hypothyroid HFpEF. Often, lncRNAs exhibit a high level of structural conservation along with conserved functions across species (Jalink et al., 2023). Thus, our results suggest that lncRNAs may play an important role in the progression and/or severity of aging-related HFpEF. We have also identified one significantly upregulated salt-inducible kinase 1 (*Sik1*) and one downregulated annexin A13 (*Anxa13*) mRNA in early (5 mo) ZSF1 lean control vs obese HFpEF in comparisons of the microarray analyses. *Sik1* has been previously shown to be involved in promoting pathologic cardiac remodeling but has not been implicated in HFpEF (Hsu et al., 2020). On the other hand, *Anxa13* has never conclusively reported to be involved in the development or progression of CVDs. Taken together, this is the first report showing dysregulation of both *Sik1* and *Anxa13* in HFpEF.

Hypertension is one of the most common co-morbidities in HFpEF patients. It is not only a modifiable risk factor but has also been found to be a critical contributor to the pathogenesis and prognosis of the disease (Tsioufis et al., 2017). Both ZSF1 lean controls and ZSF1 obese rats are hypertensive at ∼5-mo age or earlier (Riboulet et al., 2013; Nguyen et al., 2020, 2023), and we included WT controls to identify potential molecular factors influencing the genotypes. Interestingly, we found 26 up- and 21 downregulated mRNAs when comparing either 5 mo WT control and ZSF1 or 5 mo WT control and obese ZSF1 rats (Table S3). Similarly, 12 up- and 12 downregulated lncRNAs were found when comparing the above mentioned groups of rats. It is possible that these genes are associated with hypertension, an important common feature among both ZSF1 lean and obese rats. The commonalities in these ncRNAs and coding RNAs between both non-HF (lean) and HF (obese) phenotypes suggest that drugs targeting these genes may help improve both these pathological conditions. Compared to hearts of WT control, obese hearts showed GO enrichment changes predominantly in metabolic and lipid pathways. On the other hand, the ZSF1 lean control GO pathways showed changes predominantly in immune mechanisms. Similar findings were found in the KEGG analyses. Compared to both control groups, the LVs in the obese HFpEF group showed significant upregulation predominantly in lipid-based metabolic pathways. These findings suggest that metabolic/lipid factors play a larger role in HFpEF hearts alongside other comorbidities, such as hypertension, diabetes mellitus, etc. These findings may also help in identifying nodes and targets in the development of novel interventions for HFpEF.

Ang II is a critical factor in the renin−angiotensin−aldosterone pathway and excess activation of this system, in association with abnormal inflammatory processes, plays crucial roles in the development and worsening of heart failure, especially HFpEF (Gladden et al., 2018; Komajda and Lam, 2014; A et al., 2018; D'Elia et al., 2015; Feng et al., 2021; Liu et al., 2011; Mishra and Kass, 2021; Schiattarella et al., 2021; Shang et al., 2008; Shyni et al., 2021; Thorp and Filipp, 2025; Tourki and Halade, 2021; Yang et al., 2012; Zhang et al., 2024; Zhou et al., 2014, 2024). While *LOC100912003* knockdown showed significant improvement after treatment with either Ang II or LPS, knockdown of *LOC102555623* or *LOC102553944* showed improvements only in Ang II-treated cells – potentially pointing towards their divergence in modes of action. In addition, the *in vitro* results also highlight the synergistic effect of T3 (the active form of TH) with the lncRNA *LOC100912003* providing a cardioprotective effect. Furthermore, evolutionary conservation of noncoding regions has been documented in mammalian species (Necsulea et al., 2014; Hezroni et al., 2015). We identified several differentially expressed rat lncRNAs that harbored GENCODE-annotated lncRNAs in their corresponding syntenic human regions. Fourteen of the rat lncRNAs harbored a significant level of non-linear sequence similarity to their syntenic human lncRNA pairs, implying a possible evolutionary conservation of these lncRNAs or their underlying DNA. Among these, *ENSG00000274080*, *LINC01089*, *GFOD3P* and *SNHG20* have been associated with tumors (Hezroni et al., 2015; Ji et al., 2021; Yuan et al., 2019; Guo and Li, 2020; Li and Guo, 2020; Xu et al., 2021; Chen et al., 2017; Deng et al., 2024; Zhang et al., 2016). The lncRNA locus *CARINH* has been shown to be a regulator of inflammatory and immune responses (Ma et al., 2023; Cyr et al., 2025). A recent study identified that downregulation of *SNHG20* in HL-1 cardiomyocytes improved Ang II-induced cardiac fibrosis and hypertrophy, indicating a potential compensatory mechanism in our model (Li et al., 2021). Taken together, our work has uncovered key mechanisms that may be important in human aging-related hypothyroid HFpEF.

The comorbidities seen in HFpEF are considered to be secondary to systemic inflammation resulting in abnormal levels of inflammatory cytokines. In particular, metabolic inflammation or meta-inflammation is considered central to HFpEF pathophysiology (Schiattarella et al., 2021; Pugliese et al., 2023). The ZSF1 model serves as one of the best tools that embodies the clinical manifestations of HFpEF. We have recently shown that multiple inflammatory and immune-related lncRNAs are significantly impaired in the LVs of experimentally induced hypothyroid cardiac dysfunction (Rajagopalan et al., 2023). Our current study revealed that this is also observed in the hypothyroid HFpEF LVs, albeit via a different set of inflammatory and immune-related lncRNAs. Inflammatory biomarkers, including C-reactive protein (CRP), interleukin 1 beta (IL1B), tumor necrosis factor alpha (TNFA) (Mesquita et al., 2021) and its receptors, TNFR1 and TNFR2 (Putko et al., 2014), IL6 and the C-C motif chemokine ligand 2 (CCL2) (DeBerge et al., 2019), etc. are often elevated in patients with HFpEF. Chronic, low-grade, systemic inflammation might have detrimental effects on myocardial structure and function. In agreement with the previously reported association of inflammation in HFpEF, our microarray analyses of the 5-mo HFpEF group also showed multiple, significantly enriched inflammatory pathways in mRNA GO analyses. This primed our interest in identifying previously unreported inflammatory proteins in aging-related hypothyroid HFpEF.

FGF21 levels were significantly increased in the serum of the early obese HFpEF rats compared to the ZSF1 lean controls. This concurs with the clinical findings that circulating FGF21 is increased in HFpEF (Chou et al., 2016; Ianos et al., 2021). It would be valuable to further investigate its role in possible mechanisms of inflammaging, i.e. of a chronic increase in basal systemic inflammation (Dugan et al., 2023). We also found multiple targets significantly altered in late vs early HFpEF LV tissues. While FCGR3A, EphB3 and Serpin F1 protein levels decreased in 13-mo obese HFpEF compared to 5-mo obese HFpEF, levels of IL1R1 were found to be significantly increased in the 13-mo obese HFpEF compared to their 5-mo counterparts. Although IL1R1 has been implicated in aging-associated cardiomyopathy (Narayan et al., 2022), FCGR3A, EphB3 and Serpin F1 have not been shown to have any effects. This is the first report that uncovers the importance of these molecules in aging-related hypothyroid HFpEF. Moreover, given the significant downregulation of ECM-1 in all ZSF1 groups and time-points studied in this inflammation assay, it is possible that targeting this gene may be helpful to overcome both hypertension and HFpEF (early and late) or hypertension alone (reported in both genotypes). The potential interactions among the three lncRNAs, the two mRNAs and the panel of inflammation-related markers that we detected are not clear and a valuable area for future investigations.

The limitations of the study include the following. Although we used both females and males in the majority of the groups, we could only acquire males for the ZSF1 obese group of rats. Future assessment of ZSF1 obese female rats at both 5-mo and 13-mo can help understand any sex-related differences in HFpEF development and severity. Although this study does not establish a strong causative relationship, it does demonstrate that hypothyroidism is an important comorbidity factor in HFpEF and may contribute and/or worsen the pathology in association with ncRNA and inflammatory mechanisms. Since hypothyroidism was observed not only in the old HFpEF groups but also in our young HFpEF groups it is likely that the hypothyroidism is not primarily due to aging but, rather, a key factor in the HFpEF development. It would also be useful for future studies to employ next-generation RNA sequencing to identify any transcripts that may have been missed by microarray.

In summary, we have shown long-term mechanisms in aged HFpEF and hypertensive heart disease. More specifically, we have identified novel cardiac lncRNAs and inflammatory targets associated with persistent hypothyroidism in aging-related HFpEF syndrome. Treatment options for HFpEF are limited and future studies can help towards further understanding of mechanisms and development of better diagnostic and therapeutic targets for this life-threatening condition that presents with severe morbidity and mortality.

## MATERIALS AND METHODS

### Animal models

All protocols were approved by the Institutional Animal Care and Use Committee at Arkansas State University and performed in accordance with the Guidelines for the Care and Use of Laboratory Animals. Adult ZSF1 obese (HFpEF) 5-months-old (mo) rats were obtained from Charles River Labs (Wilmington, MA) and used in this study (Moreira-Costa et al., 2024; Leite et al., 2019). Previous studies have reported that ZSF1 obese rats die at around 12 months of age (Tofovic et al., 2000). In our cohort, we found that this group of rats began dying at around 13 months of age. Accordingly, we selected this as the additional (aged) timepoint for the ZSF1 obese group. Importantly, age-matched WT Wistar Kyoto (WKY) rats and ZSF1 lean rats are the widely established and standardized controls for the ZSF1 obese preclinical model (Moreira-Costa et al., 2024; Leite et al., 2019; Hamdani et al., 2013). We used both these control groups at both the 5-mo and 13-mo timepoints to maintain consistency with the literature and improve rigor. In addition, we included 20-mo timepoint in both the WT and ZSF1 lean control groups as the oldest aged groups. The total number of rats used was as follows: In the WKY group, we used *n*=19 rats in the 5-months group, *n*=13 rats in the 13-months group and *n*=8 rats in the 20-months group. In the ZSF1 lean control group, we used *n*=35 rats in the 5-months group, *n*=11 rats in the 13-months group and *n*=7 rats in the 20-months group. In the ZSF1 obese group, we used *n*=12 rats in the 5-months group and *n*=3 rats in the 13-months group.

### Cardiac ultrasound

Echocardiograms were performed to study cardiac function and related physiological assessments including heart rate, interventricular septal dimension in diastole and systole (IVSd and IVSs), left ventricular internal diameter in end-diastole and end-systole (LVIDd and LVIDs), left ventricular posterior wall diameter in diastole and systole (LVPWd and LVPWs). The rats were anesthetized with isoflurane (3% induction and 1.5% maintenance) as described before by Rajagopalan et al., (2017, 2016) and secured warm on an isothermal pad at 37°C during the procedure following the removal of chest hairs. Two-dimensional echocardiogram measurements were collected from LV short-axis views using a SonoSite M-Turbo system and an ultrasound transducer probe. Raw values were obtained from built-in software and analyzed. Percent fractional shortening (% FS) was calculated using the following formula: [(LVIDd−LVIDs)×100/LVIDd]. The percent ejection fraction was calculated as described before in multiple studies (reviewed by Sztechman et al., 2020). Two outliers were eliminated with a *z*-score value of >2.

### Terminal isolation

All rats were fully anesthetized with isoflurane anesthesia as before (Rajagopalan et al., 2023, 2017). Following assessment of body weights and blood sample collection from the apex of the heart, rats were euthanized using potassium chloride (20 mM; diastolic arrest) injection into the heart. Subsequently, heart, lung, liver and kidney tissues were dissected and quickly weighed for gravimetric analyses. The tissue samples were flash-frozen in liquid nitrogen and saved at −80°C until use. Left tibias were collected, and their lengths measured for normalization and comparison among the groups.

### Histological analyses

LV mid-sections harvested from the heart tissues above were embedded in OCT compound (Electron Microscopy Sciences, Hatfield, PA) and the frozen blocks were sectioned at a thickness of 10 μm. To measure cardiomyocyte areas, the cryosectioned slides were stained based on manufacturer’s protocols with FITC-conjugated wheat germ agglutinin (WGA; Biotium, Fremont, CA) to visualize cell boundaries. They were counterstained with 4′,6-diamidino-2-phenylindole (DAPI; MedChemExpress, Monmouth Junction, NJ) to label the nuclei. Briefly, the slides were washed to remove the OCT medium. They were then fixed in 10% Neutral Buffered Formalin (G-Biosciences, St Louis, MO) for 15 min, placed in a humidified chamber with WGA (1:500) and incubated in the dark at room temperature for 30 min. Following incubation and washes, the nuclear counterstain, DAPI (1:200) was applied. Following further incubation and washes, the sectioned slides were mounted using Mowiol^®^ 4-88 (Sigma, St. Louis, MO) and stored in the dark until imaging (Del Gaudio et al., 2023). Images were captured using a fluorescence microscope (Agilent BioTek Cytation). Random captures from various directions of each slide, including ∼48 cells per slide, were measured at 20× magnification using ImageJ (NIH, Bethesda, MD) (Schindelin et al., 2012) and averaged.

### Serum thyroid hormone assays

The collected blood was centrifuged at 3430 ***g*** for 15 min at 4°C to isolate the serum. Isolated serum samples were stored at −80°C in small aliquots until further use. Total T3 (tT3) and total T4 (tT4) serum levels were analyzed using respective Accubind enzyme-linked immuno-sorbent assay (ELISA) kits (Monobind Inc., Lake Forest, CA) with appropriate controls, following the manufacturer’s instructions. All available rats at all the age timepoints were used to analyze both tT3 and tT4 serum levels using ELISA.

### RNA isolation

Left ventricular (LV) tissues (∼60-80 mg) were homogenized in a bullet blender using manufacturer-suggested RINO lysis kits (Next Advances, Troy, NY). Total tissue RNA was isolated from homogenized LV tissues using TRIzol reagent (Invitrogen, USA) followed by Invitrogen Purelink RNA kit purification (Thermo Fisher Scientific, Scientific, Waltham, MA) following the manufacturer's recommendations. To ensure the RNA purity, an additional genomic DNA removal step was performed using the RNase-free DNase (Qiagen, Germantown, MD), and the RNA concentration and quality were initially assessed with Nanodrop (Thermo Fisher Scientific).

### Rat lncRNA/mRNA microarray

The total LV RNA from 5-mo groups was studied using the Agilent lncRNA Microarray platform (v2.0; 4×44K; Arraystar Inc., Rockville, MD) against a comprehensive and robust collection of 13,611 lncRNAs and 24,626 mRNAs. The experimenter(s) was blinded to the groups. The lncRNAs in the microarray were carefully curated using open-access transcriptomic databases, such as RefSeq, Ensembl and lncRNAdb (Arraystar Inc.) (Khalil et al., 2009; Guttman et al., 2009). Each transcript is represented by a specific exon or splice junction probe for accurate identification. To ensure hybridization quality control, standard positive control (housekeeping genes) and negative control probes were used. RNA quality and quantity were measured using a NanoDrop ND-1000 spectrophotometer and RNA integrity was assessed by standard denaturing agarose gel electrophoresis (Rajagopalan et al., 2023). The sample preparation and hybridization were performed based on the Agilent One-Color Microarray-Based Gene Expression Analysis protocol with minor modifications. Briefly, samples were amplified and transcribed into fluorescent cRNA along the entire length of the transcripts without 3′ bias utilizing a random priming method (Arraystar Flash RNA Labeling Kit). The labeled cRNAs were purified with RNeasy Mini Kit (Qiagen), and the specific activity and concentration of the fluorescently labeled cRNAs (pmol Cy3/μg cRNA) were quantified using NanoDrop ND-1000. 1 μg of each labeled cRNA was fragmented in a mix of 10× Blocking Agent and 25× Fragmentation Buffer at 60°C for 30 min. The fragmented cRNAs were diluted in 2× GE Hybridization buffer. A total of 50 μl of hybridization solution was dispensed into the gasket slide before assembly to the LncRNA Expression Microarray slide followed by incubation for 17 h at 65°C in an Agilent Hybridization Oven. Following washing and fixing of the slides, the arrays were scanned using Agilent Scanner G2505C.

To analyze the acquired array images, Agilent Feature Extraction software (version 11.0.1.1) was used. GeneSpring GX v12.1 software (Agilent Technologies) was used for quantile normalization (Bolstad et al., 2003) and subsequent data processing. Subsequently, after batch effects were removed using COMBAT, low-intensity lncRNAs and mRNAs were filtered. Gene Set Enrichment Analysis (GSEA) module and GseaPreRanked modules were used to perform GSEA analysis of mRNAs. At least three samples of lncRNAs were analyzed using the GseaPreRanked module by ranking the Pearson correlation coefficient between one of the selected lncRNAs (ten most differentially expressed upregulated lncRNAs and ten downregulated lncRNAs) and all mRNAs. All arrays passed multiple quality control tests.

The statistically significant differentially expressed lncRNAs and mRNAs were identified by using Fold-Change filtering between samples and Volcano filtering between groups. R software was used for Hierarchical Clustering. The thresholds were kept at a fold change ≥1.5 (*P*<0.05). The fold change between log2 transformed normalized intensities for, e.g. *norm1* compared with *norm0*, can be calculated as the fold change =2^norm1−norm0^. The initial microarray analyses and data visualization with box plots, scatter plots, volcano plots and heatmaps contained targets only with *P*-values of <0.05 but were not filtered for false discovery rates (FDRs). Therefore, we further refined the data and presented the scatter plots only with targets satisfying both *P*<0.05 and FDR<0.05 for improved reliability. For identification of differentially expressed mRNAs with statistical significance, fold-change analysis was performed; cut-offs were, again, set at a fold change ≥1.5 (*P*<0.05) and an FDR<0.05. The lncRNA−mRNA association was defined by the presence of a statistically significant (*P*<0.05, FDR<0.05) differentially expressed lncRNA region in <200 kb chromosomal vicinity of a significantly (*P*<0.05, FDR<0.05) altered mRNA region. Gene Ontology (GO) and Kyoto Encyclopedia of Genes and Genomes (KEGG) pathway analyses were performed on differentially expressed mRNAs. topGO package in the R environment was used for GO enrichment analyses, statistical computing and graphics. Fisher's exact test was used for KEGG and GO pathway analysis. The datasets are presented in Tables S1-S6.

### Quantitative real-time PCR

Based on the top hits from the lncRNA microarray, we further investigated the expression patterns of select, significant lncRNA targets (expanded later) at all possible and available timepoints using real-time quantitative PCR (RT-qPCR). First, 1 µg of isolated RNA was converted to cDNA using the RT^2^ First Strand synthesis kit (Qiagen), following the manufacturer’s instructions. qRT-PCR was performed using iTaq universal SYBR Green mastermix (BioRad, Hercules, CA) in CFX384 (BioRad). All the primers were designed using the PrimerQuest tool (IDT, Coralville, IA). Normalization was performed with glyceraldehyde 3-phosphate dehydrogenase (*GAPDH*), ribosomal protein lateral stalk subunit P1 (*Rplp1*) and beta 2-microglobulin (*B2m*) genes. Following the removal of low-quality samples with low quantification cycle (Cq) values and low-quality melting curves, automated data analysis was performed with CFX Maestro software (BioRad,) using the default 2^−ΔΔCT method. A fold change of at least ±1.5 and *P*<0.05 was considered significant. The results were expressed and plotted as relative fold change±standard error.

### Identification of human lncRNAs in genomic regions that are syntenic to those that harbor differentially expressed rat lncRNAs

To determine whether the genomic regions surrounding differentially expressed rat lncRNAs harbor GENCODE-annotated lncRNAs in their syntenic human regions, we carried out the following analyses (Mudge et al., 2025). We added 10 kb to the Rnor_5.0 genome assembly (rn5) start and end coordinates of each rat lncRNA, and used the University of California, Santa Cruz (UCSC) LiftOver (https://genome.ucsc.edu/cgi-bin/hgLiftOver) to identify putative syntenic regions in the human genome build 38 (hg38) genome assembly (Perez et al., 2025; Hinrichs et al., 2006). All lifted-over segments that had at least one set of human coordinates spanning at least 10% of the length of searched rat coordinates were then searched for GENCODE lncRNAs using GenomicRanges (https://bioconductor.org/packages/release/bioc/html/GenomicRanges.html; Lawrence et al., 2013). For each rat segment searched, we retained only the longest human lifted-over segment in hg38 for subsequent evaluation. Human lncRNAs paired with their corresponding rat lncRNAs were then compared using SEquence Evaluation through k-mer Representation (SEEKR; https://github.com/CalabreseLab/seekr) (Li et al., 2024; Kirk et al., 2018). SEEKR was performed using a *k*-mer length of *k*=4, log-transformed and against the background set of human GENCODE 47 (https://www.gencodegenes.org/human/release_47.html) canonical non-redundant lncRNAs that are >500 nucleotides long.

### *In-vitro* assessment of the aging-related HFpEF-associated lncRNAs

In order to identify the physiological relevance of the key lncRNAs detected via microarray and qPCR in cardiac cells, rat cardiomyoblast H9c2 cells were obtained from the ATCC and authenticated for cardiac genes. H9c2 cells were used and cultured in DMEM, high glucose (Thermo Fisher Scientific), 10% FBS (Thermo Fisher Scientific), 1% Penicillin-Streptomycin (Sigma, St. Louis, MO) to 80-90% confluency. Cells were then trypsinized with 0.25% trypsin-EDTA (Thermo Fisher Scientific), counted in Countess™ 3 Automated Cell Counter and 20,000 cells were plated in 96 well plates in quadruplicates. The cells were then transfected with the siRNAs (Synbio Technologies, Monmouth Junction, NJ) using RNAiMax (Thermo Fisher Scientific) following manufacturers’ protocols. After 48 h, the transfected cells were treated for 24 h with either 100 nM angiotensin II (Ang II; MedChemExpress) or 5 μg/ml lipopolysaccharide (LPS; MedChemExpress) with or without 10 μM T3 (Sigma, St. Louis, MO). The doses were selected based on prior studies (A et al., 2018; D'Elia et al., 2015; Feng et al., 2021; Liu et al., 2011; Mishra and Kass, 2021; Sacripanti et al., 2018; Schiattarella et al., 2021; Shang et al., 2008; Shyni et al., 2021; Thorp and Filipp, 2025; Tourki and Halade, 2021; Yang et al., 2012; Zhang et al., 2024; Zhou et al., 2014, 2024). The cell viability was assessed with an MTT assay kit (Cayman Chemicals, Ann Arbor, MI) following the manufacturer's instructions.

### Detection of altered inflammatory factors associated with HFpEF

Both **s**erum and LV tissue lysates from early (5 mo) WT control, ZSF1 lean and ZSF1 obese, as well as late (13 mo) ZSF1 obese were used to identify key alterations in inflammatory factors. The tissue lysates were prepared following standard protocol. Briefly, LV tissue sections were homogenized in 300 μl RIPA buffer and Protease Inhibitor cocktails with a bullet blender using the manufacturer-suggested RINO lysis kits (Next Advance Troy, NY). The tissue lysates were then agitated at 4°C for 2 h and the supernatants were collected following centrifugation at 16,000 ***g*** for 20 min at 4°C. The protein quantifications were performed using a Pierce BCA protein kit, following the manufacturer's guidelines. Subsequently, diluted tissue lysates with a final protein concentration of 25 μg and 1:2 diluted serum samples (with manufacturer-provided sample diluent to a final volume of 100 µl) were used for the streptavidin/biotin-based G-Series Rat Cytokine Array 9 (Ray Biotech, Peachtree Corners, GA), following the manufacturer’s instructions. The slides were dried using compressed nitrogen gas flow followed by fluorescent imaging and densitometry analyses.

Biotinylated IgG was used as positive control, and for signal normalization, detection monitoring and image orientation. The empty spots on the array were used as negative control or background. The raw data were normalized by RayBiotech analysis using *X(nY)=X(Y)×P1/P(Y)*, with *P1* referring to the average signal density of the positive control spots on the reference array, *P(Y)* referring to the average signal density of the positive control spots on array Y, *X(Y)* referring to the signal density for a particular spot on array for sample ‘Y’, *X(nY)* referring to the normalized value for that particular spot ‘X’ on array for sample ‘Y’. The extracted raw data from quadruplicated assays of 40 distinct inflammatory target proteins were grouped and statistically analyzed in GraphPad Prism, and using one-way ANOVA with Tukey’s multiple comparison tests.

### Statistical analyses

The data and statistical analysis for microarray have already been described above. All results were expressed as means±standard deviation unless noted otherwise. Statistical data analyses were performed with GraphPad Prism (versions 9.5.1 and 8.0.2) software. Analyses of groupwise comparisons were carried out using one-way or two-way analysis of variance (ANOVA) with Tukey's multiple comparisons tests or *t*-test as appropriate. *P*<0.05 was considered statistically significant.

## Supplementary Material

10.1242/dmm.052207_sup1 Supplementary information

Table S1. LncRNA Differential Expression Analysis

Table S2. mRNA Differential Expression Analysis

Table S3. mRNA GO_Enrichment

Table S4. mRNA KEGG Pathway Enrichment Analyses

Table S5. LincRNAs associated coding gene data table

Table S6. Human LncRNA Sequence Similarity
