# Supplementary material for: Noncoding and coding mechanisms of aging-related heart failure with preserved ejection fraction associated with thyroid dysfunction
Source: Dis Model Mech. 2025 Nov 25;18(11):dmm052207. doi: 10.1242/dmm.052207 (PMC12690541; doi:10.1242/dmm.052207)
Supplement: Supplementary information [file dmm-18-052207-s1.pdf]

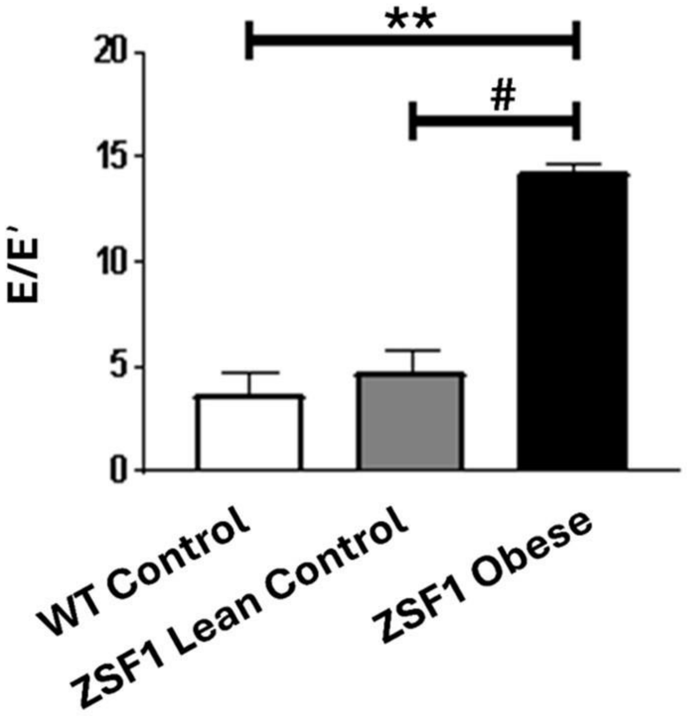


**Fig. S1. In early HFpEF rats, echocardiography showed significantly elevated E/E' indicating diastolic dysfunction.** All values are analyzed using one-way ANOVA and presented as means ± standard deviation; E/E': ratio of early mitral inflow velocity and mitral annular early diastolic velocity; WT: Wild Type; $^{**}p<0.01$; $^{\#}p<0.05$.

Disease Models & Mechanisms • Supplementary information

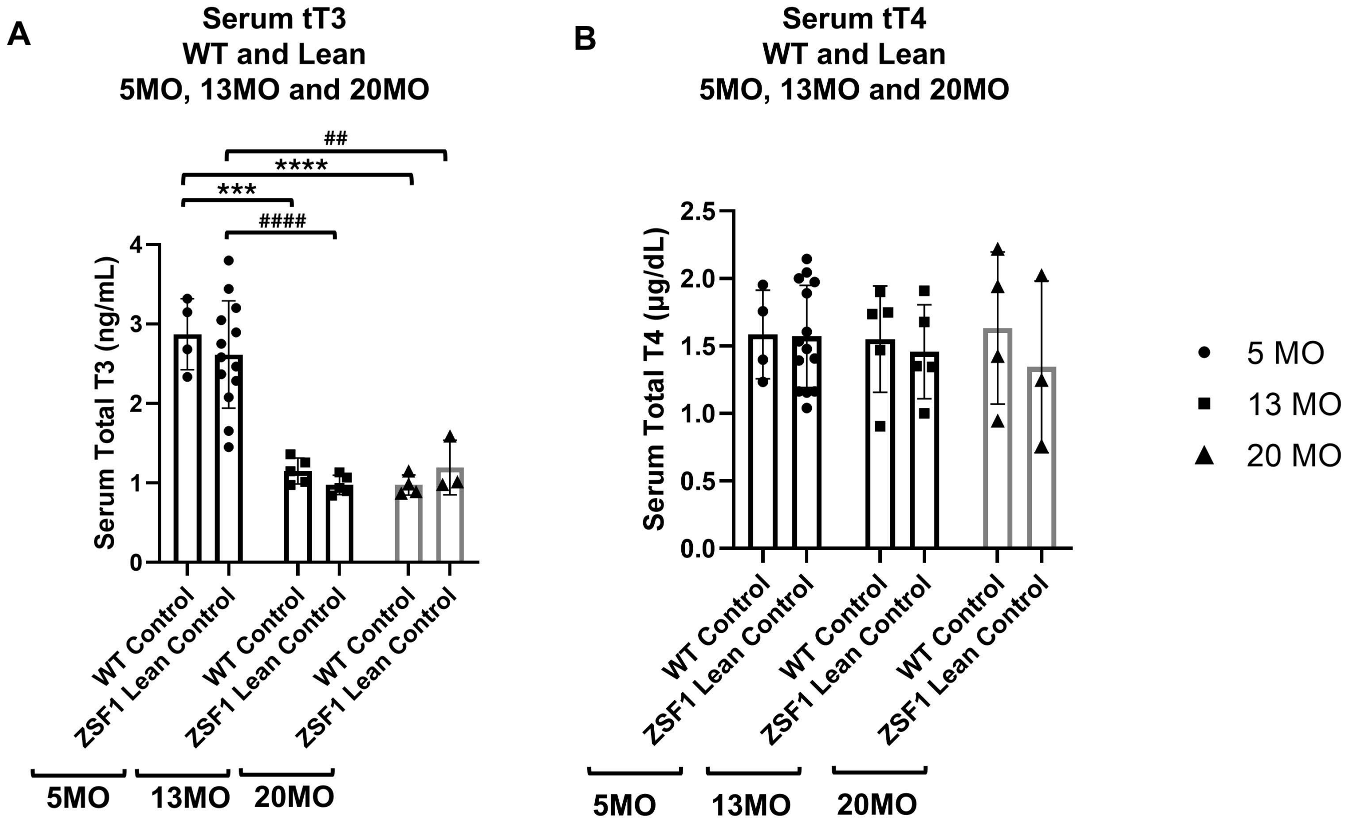


**Fig. S2. Serum thyroid hormone levels (Females).** Serum **(A)** total T3 and (**B**) total T4 levels in 5 mo, 13 mo, and 20 mo female WT control and ZSF1 lean control groups. All values are analyzed using Two-way ANOVA with Tukey's multiple comparison test. and presented as means ± standard deviation; WT: Wild Type; T4: Thyroxine; T3: Triiodothyronine; ***$p<0.001$, ****$p<0.0001$ vs 5 mo WT control; $^{\#\#}p<0.01$, $^{\#\#\#\#}p<0.0001$ vs 5 mo ZSF1 lean control.

Disease Models & Mechanisms • Supplementary information

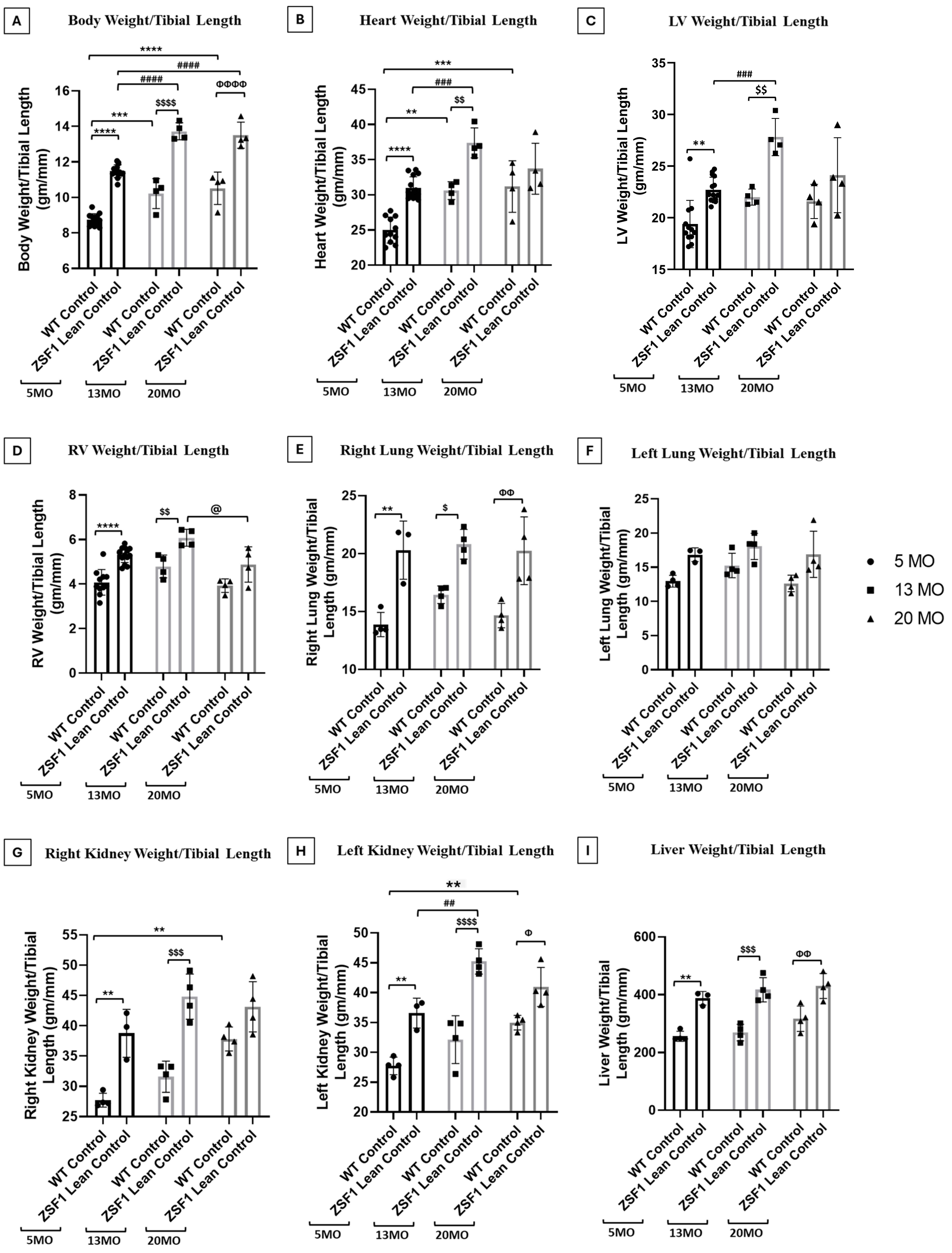
A
Body Weight/Tibial Length
Body Weight/Tibial Length (gm/mm)
B
Heart Weight/Tibial Length
Heart Weight/Tibial Length (gm/mm)
C
LV Weight/Tibial Length
LV Weight/Tibial Length (gm/mm)
D
RV Weight/Tibial Length
RV Weight/Tibial Length (gm/mm)
E
Right Lung Weight/Tibial Length
Right Lung Weight/Tibial Length (gm/mm)
F
Left Lung Weight/Tibial Length
Left Lung Weight/Tibial Length (gm/mm)
G
Right Kidney Weight/Tibial Length
Right Kidney Weight/Tibial Length (gm/mm)
H
Left Kidney Weight/Tibial Length
Left Kidney Weight/Tibial Length (gm/mm)
I
Liver Weight/Tibial Length
Liver Weight/Tibial Length (gm/mm)
WT Control
ZSF1 Lean Control
5MO
13MO
20MO
5 MO
13 MO
20 MO

**Fig. S3. Gravimetrics in males (normalized by tibial length - 5 mo, 13 mo, 20 mo - WT control and ZSF1 lean control) - (Male). (A)** Body weight/Tibial length; **(B)** Heart weight/Tibial length; **(C)** LV weight/Tibial length; **(D)** RV weight/Tibial length; **(E)** Right lung weight/Tibial length; **(F)** Left lung weight/Tibial length; **(G)** Right kidney weight/Tibial length; **(H)** Left kidney weight/Tibial length and **(I)** Liver weight/Tibial length. All values are analyzed using Two-way ANOVA with Tukey's multiple comparison test. and presented as means ± standard deviation; WT: Wild-type, LV: Left ventricle, RV: Right ventricle weight; $^{**}p<0.01$, $^{***}p<0.001$, $^{****}p<0.0001$ vs 5 mo WT control; $^{\#\#}p<0.01$, $^{\#\#\#\#}p<0.0001$ vs 5 mo ZSF1 lean control; $^{\$}p<0.05$, $^{\$\$}p<0.01$, $^{\$\$\$}p<0.001$, $^{\$\$\$\$}p<0.0001$ vs 13 mo WT control; $^{@}p<0.05$ vs 13 mo ZSF1 lean control; $^{\Phi}p<0.05$, $^{\Phi\Phi}p<0.01$, $^{\Phi\Phi\Phi\Phi}p<0.0001$ vs 20 mo WT control.

Disease Models & Mechanisms • Supplementary information

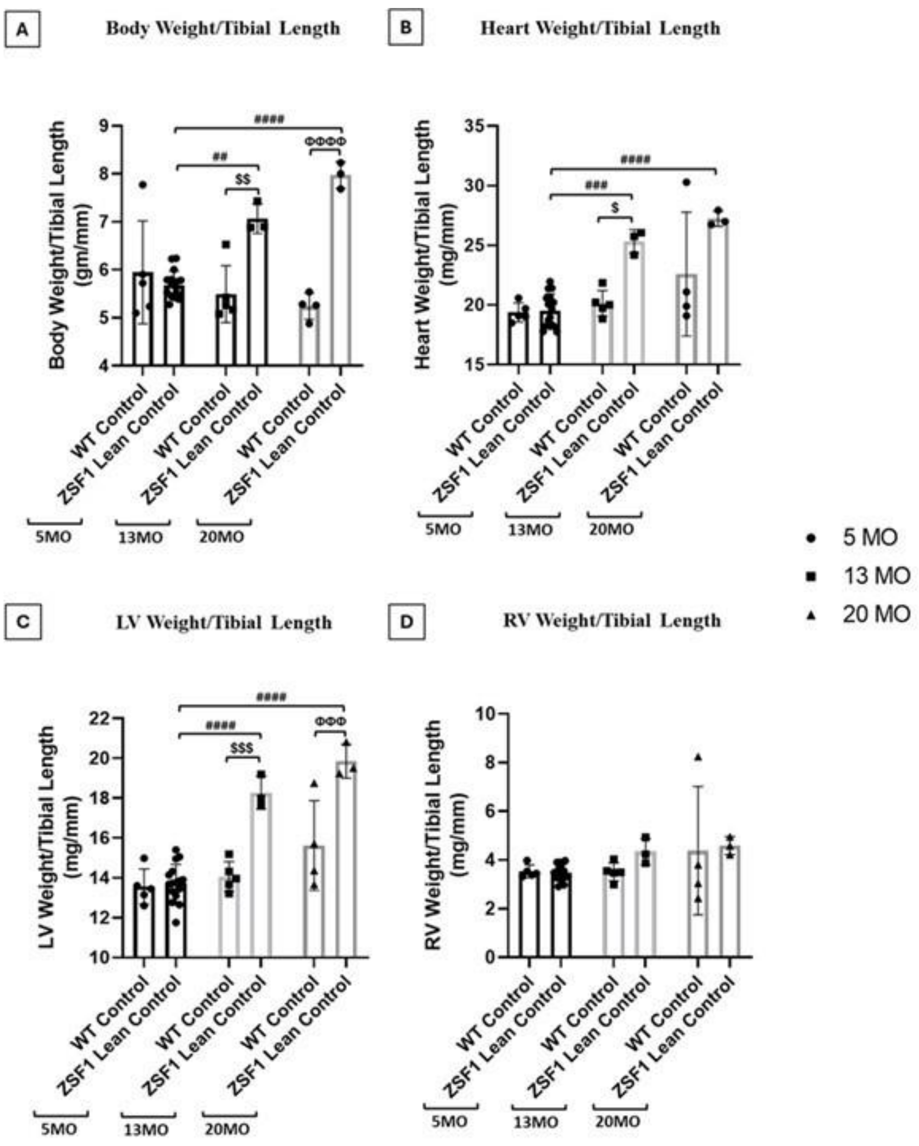


**Fig. S4. Gravimetric analyses (Female) of 5 mo, 13 mo, and 20 mo WT control and ZSF1 lean control rats (A)** Body weight/Tibial Length **(B)** Heart weight/Tibial Length **(C)** LV weight/Tibial Length and **(D)** RV weight/Tibial Length. All values are analyzed using Two-way ANOVA with Tukey's multiple comparison test. and presented as means ± standard deviation; WT: Wild-type, LV: Left ventricle, RV: Right ventricle weight; $^{\#\#}p<0.01$, $^{\#\#\#}p<0.001$, $^{\#\#\#\#}p<0.0001$ vs 5 mo ZSF1 lean control; $^{\$}p<0.05$, $^{\$\$}p<0.01$, $^{\$\$\$}p<0.001$ vs 5 mo ZSF1 obese (HFpEF); $^{\Phi\Phi\Phi}p<0.001$, $^{\Phi\Phi\Phi\Phi}p<0.0001$ vs 20 mo WT control.

Disease Models & Mechanisms • Supplementary information

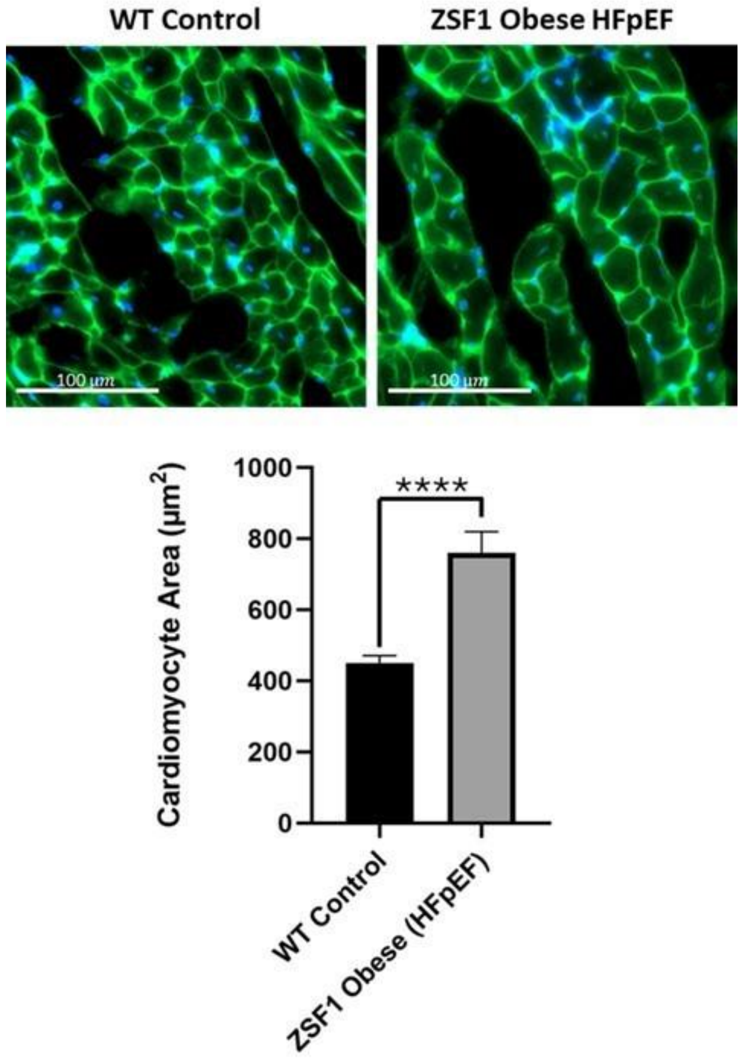


**Fig. S5. Histology shows significant cardiomyocyte hypertrophy in ZSF1 Obese rat hearts.** Cryosections of WKY wild type (WT) and ZSF1 Obese 5 mo male rat hearts were stained with Wheat Germ Agglutinin (green) and DAPI (blue for nuclei). 20X images are shown. The cardiomyocyte area was measured using Image J. Statistical analyses were performed using t-test and data are presented as mean +/- standard error. N=3/group; ****p<0.0001.

Disease Models & Mechanisms • Supplementary information

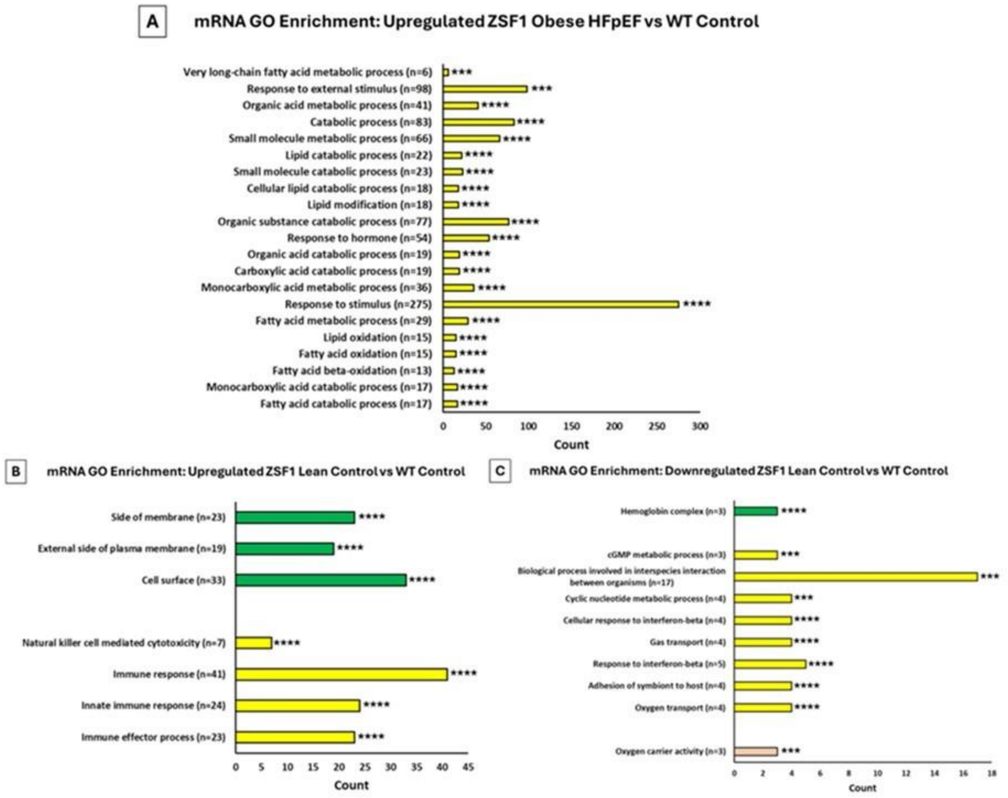


**Fig. S6. GO enrichment analyses of mRNAs**: **(A-C)** Significantly enriched GO terms ($p<0.05$ and $FDR<0.05$) in **(A)** Upregulated in ZSF1 obese vs WT control; **(B)** Upregulated in ZSF1 lean vs WT control; **(C)** Downregulated in ZSF1 lean control vs WT control; GO: Gene Ontology; ***$p<0.001$, ****$p<0.0001$.

Disease Models & Mechanisms • Supplementary information

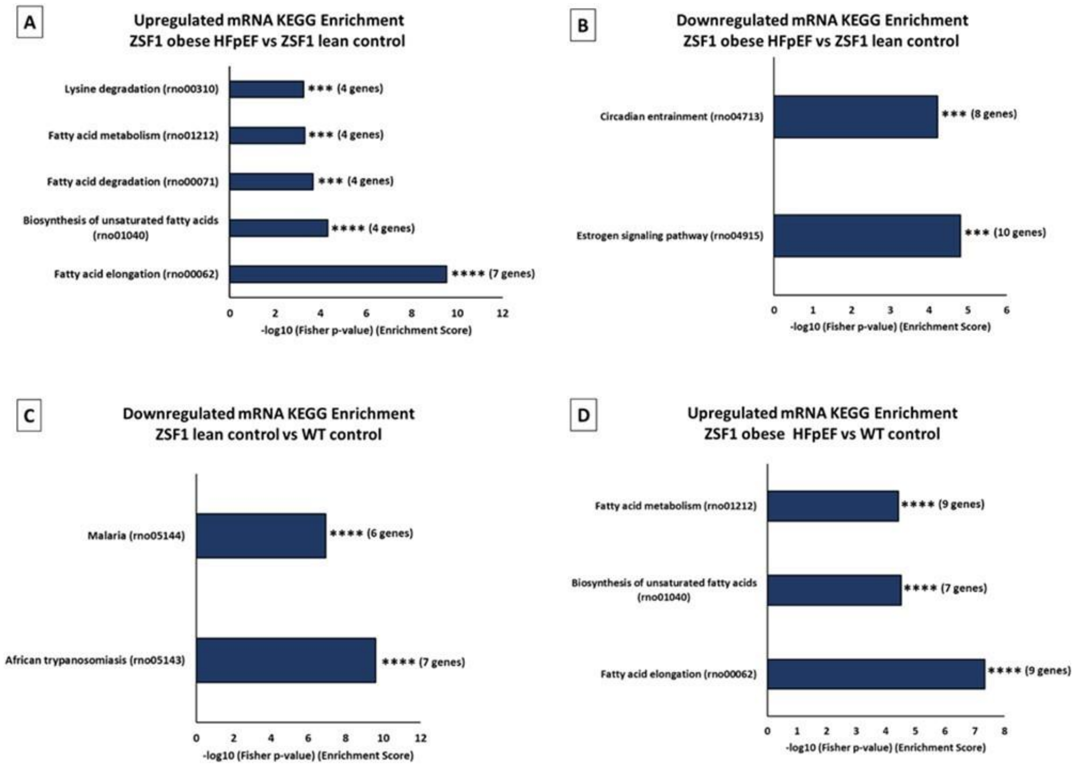


**Fig. S7. KEGG Enrichment analyses of mRNAs. (A-D)** Significantly enriched KEGG pathways ($p<0.05$ and FDR$<0.05$) in **(A)** Upregulated in ZSF1 obese vs lean control; **(B)** Downregulated in ZSF1 obese vs lean control; **(C)** Downregulated in ZSF1 lean control vs WT control; **(D)** Upregulated in ZSF1 obese vs WT control; KEGG: Kyoto Encyclopedia of Genes and Genomes; ***$p<0.001$, ****$p<0.0001$.

**Table S1. LncRNA Differential Expression Analysis**

Available for download at

https://journals.biologists.com/dmm/article-lookup/doi/10.1242/dmm.052207#supplementary-data

**Table S2. mRNA Differential Expression Analysis**

Available for download at

https://journals.biologists.com/dmm/article-lookup/doi/10.1242/dmm.052207#supplementary-data

**Table S3. mRNA GO_Enrichment**

Available for download at

https://journals.biologists.com/dmm/article-lookup/doi/10.1242/dmm.052207#supplementary-data

**Table S4. mRNA KEGG Pathway Enrichment Analyses**

Available for download at

https://journals.biologists.com/dmm/article-lookup/doi/10.1242/dmm.052207#supplementary-data

**Table S5. LincRNAs associated coding gene data table**

Available for download at

https://journals.biologists.com/dmm/article-lookup/doi/10.1242/dmm.052207#supplementary-data

**Table S6. Human LncRNA Sequence Similarity**

Available for download at

https://journals.biologists.com/dmm/article-lookup/doi/10.1242/dmm.052207#supplementary-data
